# Problems with the outcome measures in randomized controlled trials of traditional Chinese medicine in treating chronic heart failure caused by coronary heart disease: a systematic review

**DOI:** 10.1186/s12906-021-03378-z

**Published:** 2021-08-31

**Authors:** Jiayuan Hu, Ruijin Qiu, Chengyu Li, Min Li, Qianqian Dai, Shiqi Chen, Chen Zhao, Hongcai Shang

**Affiliations:** 1 Beijing Hospital of Traditional Chinese Medicine Affiliated to Capital Medical University, Beijing, 100010 China; 2 Dongzhimen Hospital Affiliated to Beijing University of Chinese Medicine, Beijing, 100700 China; 3 Institute of Basic Research in Clinical Medicine, China Academy of Chinese Medical Sciences, Beijing, 100700 China

**Keywords:** Chronic heart failure, Coronary heart disease, Traditional Chinese medicine, Randomized controlled trial, Outcome measures, Clinical end points, Reporting quality

## Abstract

**Background:**

Traditional Chinese medicine (TCM) has gained widespread application in treating chronic heart failure (CHF) secondary to coronary heart disease (CHD). However, the sound clinical evidence is still lacking. Corresponding clinical trials vary considerably in the outcome measures assessing the efficacy of TCM, some that showed the improvement of clinical symptoms are not universally acknowledged. Rational outcome measures are the key to evaluate efficacy and safety of each treatment and significant elements of a convincing clinical trial. We aimed to summarize and analyze outcome measures in randomized controlled trials (RCTs) of TCM in treating CHF caused by CHD, subsequently identify the present problems and try to put forward solutions.

**Methods:**

We systematically searched databases including Embase, PubMed, Cochrane Library, CBM, CNKI, VIP and Wanfang from inception to October 8, 2018, to identify eligible RCTs using TCM interventions for treating CHF patients caused by CHD. Cochrane Database of Systematic Reviews (CDSR) was searched to include Cochrane systematic reviews (CSRs) of CHF. Two authors independently assessed the risk of bias of the included RCTs according to the Cochrane Handbook. Outcome measures of each trial were extracted and analyzed those compared with the CSRs. We also evaluated the reporting quality of the outcome measures.

**Results:**

A total of 31 RCTs were included and the methodology quality of the studies was generally low. Outcome measures in these RCTs were mortality, rehospitalization, efficacy of cardiac function, left ventricular ejection fraction (LVEF), 6 min’ walk distance (6MWD) and Brain natriuretic peptide (BNP), of which mortality and rehospitalization are clinical end points while the others are surrogate outcomes. The reporting rate of mortality and rehospitalization was 12.90% (4/31), the other included studies reported surrogate outcomes. As safety measure, 54.84% of the studies reported adverse drug reactions. Two trials were evaluated as high in reporting quality of outcomes and that of the other 29 studies was poor due to lack of necessary information for reporting.

**Conclusions:**

The present RCTs of TCM in treating CHF secondary to CHD did not concentrate on the clinical end points of heart failure, which were generally small in size and short in duration. Moreover, these trials lacked adequate safety evaluation, had low quality in reporting outcomes and certain risk of bias in methodology. For objective assessment of the efficacy and safety of TCM in treating CHF secondary to CHD, future research should be rigorous designed, set end points as primary outcome measures and pay more attention to safety evaluation throughout the trial.

**Supplementary Information:**

The online version contains supplementary material available at 10.1186/s12906-021-03378-z.

## Introduction

Heart Failure (HF), a clinical syndrome of the dysfunction of ventricular filling or ejection led by the abnormality of cardiac structure and function, affects about 26 million people around the world [1]. The prevalence of HF is 1–2% of the adult population in developed countries [2, 3] and in China there are about 4.5 million patients of HF [4]. Although the treatment of Chronic Heart Failure (CHF) has made great progress, the mortality and rehospitalization of CHF remain high, only half of patients could survive for more than 5 years [5, 6]. The mortality of hospitalized patients with CHF was 4.1% according to the China-HF registry study [7]. From 2000 to 2010, the cardiovascular hospitalization of CHF has not decreased [8].

Coronary Heart Disease (CHD) is the first cause of CHF among all the primary diseases [9, 10], so that the prevention and treatment of CHF caused by CHD is a significant part of cardiovascular health decisions. Traditional Chinese medicine has been widely used to treat all kinds of CHF which could effectively reduce the levels of N-terminal pro-brain natriuretic peptide (NT-proBNP) [11]. However, evidence from TCM clinical trials has not been universally acknowledged in the international medical system nor been included in clinical practice guidelines. The available randomized controlled trials (RCTs) are suboptimal with diverse outcome measures, many of which only showed the improvement of symptoms. To understand the status quo of outcome measures in RCTs of TCM in treating CHF caused by CHD, we conducted a systematic review to evaluate the outcome measures, identify relevant problems and try to put forward solutions.

## Methods

### Eligibility criteria

We included RCTs meeting the following criteria: (1) performed in CHF patients with CHD as primary disease (2) assessing TCM treatment compared with a control group (without restriction). Exclusion criteria were: (1) duplicate publication (2) studies without full text.

### Information sources

Electronic databases including Embase, PubMed, Cochrane Central Register of Controlled Trials (CENTRAL) and China National Knowledge Infrastructure (CNKI), Chinese Scientific Journal Database (VIP), Wanfang and Chinese Biomedicine Literature Database (CBM) were searched from inception to October 8, 2018. Bibliographies of selected articles were also consulted in search of additional trials not detected in the initial searches.

We also searched Cochrane Database of Systematic Reviews (CDSR) to collect Cochrane systemic reviews (CSRs) of CHF for comparative analysis.

### Search

We conducted a systematic search. “Medicine, Chinese Traditional [MeSH]”, “Heart Failure [MeSH]”, “Randomized Controlled Trial [Publication Type]” were applied as search terms and free words were used according to the characteristics of each database. The detailed search strategy was shown in Additional file 1.

### Study selection

Two reviewers (JY H and RJ Q) independently selected the eligible studies, first through title and abstract and afterwards through the full text. Any disagreements of the selection period were discussed, and if the discussion could not resolve the problem, we consulted the third author (M L) and reached consensus.

### Data collection process and data items

Reviewers JY H and CY L independently extracted information of the studies using a standardized data extraction form including the first author, year of publication, disease type, sample size, interventions in the treatment and control group and outcome measures.

### Risk of bias in individual studies

We used the Cochrane Handbook for Systematic Reviews of Interventions version 5.1.0 [12] to assess the risk of bias of the included RCTs. Two reviewers (JY H and RJ Q) individually assessed the risk of bias and if there existed any disagreements, we resolved it through discussion with a third author (HC S).

### Summary measures

We calculated the reporting rate of each outcome measure in the included RCTs and conducted comparative analysis with that in the CSRs of CHF. On account of the aim to analyze outcome measures, we did not synthesize data of the trials nor conduct a meta-analysis.

### Additional analyses

Two authors (JY H, RJ Q) independently evaluated the reporting quality of outcome measures in the included RCTs based on the Management of Otitis Media with Effusion in Cleft Palate (MOMENT) criteria [13], considering the following 6 items:

1. Is the primary outcome clearly stated?
2. Is the primary outcome clearly defined so that another researcher would be able to reproduce its measurement? Where appropriate, this should include clear descriptions of time points, the person measuring the outcome, how the outcome was measured (for example, tools and methods used) and where the outcome was measured.
3. Are the secondary outcomes clearly stated?
4. Are the secondary outcomes clearly defined?
5. Do the authors explain the use of the outcomes they have selected?
6. Are methods used to enhance the quality of outcome measurement (for example, repeated measurement, training) if appropriate?

## Results

### Study selection

**Fig. 1:**
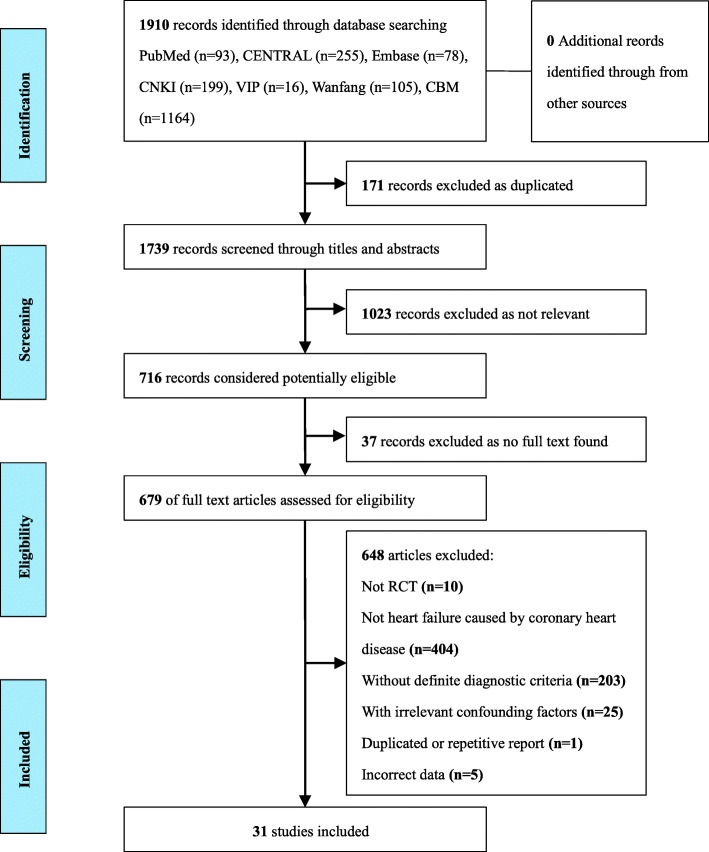
Flowchart of study selection

We identified 1910 records from the seven databases. Firstly we excluded 171 duplicated records and 1023 records through titles and abstracts. Then 679 full-text articles were assessed for eligibility and 648 articles were eliminated for the reasons shown in Fig. 1. Finally, we included and analyzed 31 RCTs [14–44] in the review. We also screened sixteen CSRs of CHF [45–60].

### Study characteristics

Thirty-one included studies were all conducted in China and 29 were published in Chinese, two were published in English [39, 40]. The main information of each study is shown in Table 1 and the information of 16 CSRs of CHF in Table 2.

**Table 1:** Information of included studies (*n* = 31)

Study ID	Sample (T/C)	Disease	Interventions (T/C)	Duration	Outcomes
Junxian Qi 2010 [14]	30/30	CHF in CHD	QiShen YiQi dropping pill + RT / RT	1 month	③
Dong Wang 2010 [15]	89/76	CHF in CHD	QiShen YiQi dropping pill + RT / RT	1 year	②③④
Lanfang Ren 2017 [16]	58/42	CHF in CHD after MI	QiShen YiQi dropping pill + RT / RT	30 days	③④⑤⑥
Zhitian Zhou 2005 [17]	30/30	CHF in CHD	ShenFu Injection + RT / RT	2 weeks	③
Changling Yuan 2012 [18]	82/80	CHF in CHD	YiQi FuMai Injection + RT / RT	1 month	④⑤
Litao Qu 2017 [19]	60/60	CHF in CHD	SanXianQiangXin decoction + RT / RT	4 weeks	③④⑤
Qing Zou 2012 [20]	50/50	CHF in CHD	ShenQiQiangXin decoction + RT / RT	6 months	①②③④⑤
Yunyou Cheng 2012 [21]	60/60	CHF in CHD	GuanXinKang capsule + RT / RT	2 weeks	③④⑤
Lihong Gong 2012 [22]	140/140	CHF in CHD	QiangXinTongMai granule + RT / RT	6 months	①②⑤
Junli Lu 2012 [23]	57/56	CHF in CHD	BaoYuan decoction + RT / RT	6 weeks	③
Xu Gu 2003 [24]	68/66	CHF in CHD	Astragalus injection + RT / RT	4 weeks	③④
Dongmin Liu 2011 [25]	34/34	CHF in CHD	JiaWeiLinGuiZhuGan decoction + RT / RT	4 weeks	③④⑥
Hua Zhou 2007 [26]	27/23	CHF with angina pectoris	LuHongQiangXinKuoMai granule + RT / RT	2 weeks	③
Renkui Lai 2015 [27]	30/30	CHF in CHD	NuanXin capsule + RT / RT	3 months	①③④⑤
Na Lin 2017 [28]	50/50	CHF in CHD	PingChuanGuBen decoction /RT	8 weeks	②⑤
Deyu Zhao 2011 [29]	49/49	CHF in CHD in elderly	SanShenYiXin decoction + RT / RT	15 days	③④⑥
Wei Zhang 2010 [30]	39/38	CHF in CHD	YangXinShi tablet /lotensin + metoprolol	4 weeks	③④⑤
He Li 2013 [31]	30/30	LVDD in CHD	JiaWeiShengXian decoction + RT / RT	8 weeks	③⑥
Hao Huang 2006 [32]	30/30	CHF in CHD	YiQiTongLuoLiShui formula + RT / RT	4 weeks	③
Zhen Yang 2016 [33]	43/43	CHF in CHD	WenYangHuoXueLiShui formula + RT / RT	7 months	③④⑤
Xinping Niu 2015 [34]	30/30	CHF after MI	YiQiYangYin formula + RT / RT	3 months	③④
Jie Xu 2005 [35]	40/30	LVDD in CHD	YiShenShuXin pill / diltiazem	4 weeks	③
Haitao Liu 2003 [36]	76/74	CHF in CHD	YiQiHuoXueWenYangLiShui formula + RT / RT	3 weeks	③
Youhe Ma 2001 [37]	68/45	CHF in CHD	QiangXin decoction / RT	2 weeks	③
Yuan Liu 1996 [38]	32/20	Cardiac dysfunction in CHD	YiQiHuoXue formula / nifedipine	2 weeks	③
Shaoxiang Xian 2016 [39]	114/114	CHF in CHD	ShenMai injection + RT / RT	1 week	④⑤
Liangtao Luo 2014 [40]	110/109	CHF in CHD	KangRenTang Chinese herb granule + RT / KangRenTang placebo granule + RT	4 weeks	①③
Zhanfeng Zhang 2018 [41]	36/36	Severe CHF in CHD	QiangXin decoction + RT / RT	12 weeks	③④
Songyu Zhang 2018 [42]	40/40	CHF in CHD with VPB	WenXin granule + RT / RT	3 months	④⑤
Yongzhi Wang 2018 [43]	55/55	CHF in CHD	YiQiQuYu formula+ RT / RT	14 days	③⑤⑥
Junfang Lv 2018 [44]	53/53	CHF in CHD	JiaWeiSanRen decoction+ RT / RT	not mentioned	③④⑤

Notes: ①mortality ②rehospitalization ③efficacy of cardiac function ④left ventricular ejection fraction (LVEF) ⑤6 min’ walk distance (6MWD) ⑥Brain natriuretic peptide (BNP); *T* Treatment group, *C* Control group, *CHD* Coronary heart disease, *CHF* Chronic heart failure, *LVDD* Left ventricular diastolic dysfunction, *MI* Myocardial infarction, *VPB* Ventricular premature beat, *RT* Routine treatment

**Table 2:** Information of CSRs of CHF (*n* = 16)

CSRs	Disease	Outcomes
Guo R 2008 [45]	CHF	①②⑩
Ngo K 2010 [46]	Anaemia in CHF	①③⑥⑦⑧⑨⑩
Heran BS 2012 [48]	CHF	①②⑤⑩
Takeda A 2012 [47]	CHF	①③④
Hood 2014 [49]	CHF in sinus rhythm	①③④
Lip GY 2014 [50]	CHF in sinus rhythm	①②
Madmani ME 2014 [51]	CHF	①②③⑥⑦⑧⑩
Taylor RS 2014 [52]	CHF	①③⑤⑥
Driscoll A 2015 [53]	CHF	①③⑤⑩
Inglis SC 2015 [54]	CHF	①③⑤⑥
Alabed S 2016 [55]	CHF	①⑦⑩
Fisher SA 2016 [56]	CHF	①②③⑦⑩
Martí-Carvajal AJ 2016 [57]	CHF in with Chagas cardiomyopathy	①③④⑥⑩
McLellan J 2016 [58]	CHF	①③④⑤⑥⑩
Shantsila E 2016 [59]	CHF in sinus rhythm	①②⑩
Martin N 2018 [60]	CHF	①③④⑥

Notes: ①all-cause mortality ②cardiovascular events ③heart failure (HF) hospitalization ④cardiovascular mortality ⑤all-cause hospitalization ⑥evaluation of quality of life ⑦left ventricular ejection fraction (LVEF) ⑧classification of cardiac function ⑨Brain natriuretic peptide (BNP) ⑩(adverse drug reactions) ADRs; *CHF* Chronic heart failure, *CSRs* Cochrane systematic reviews

### Risk of bias within studies

**Fig. 2:**
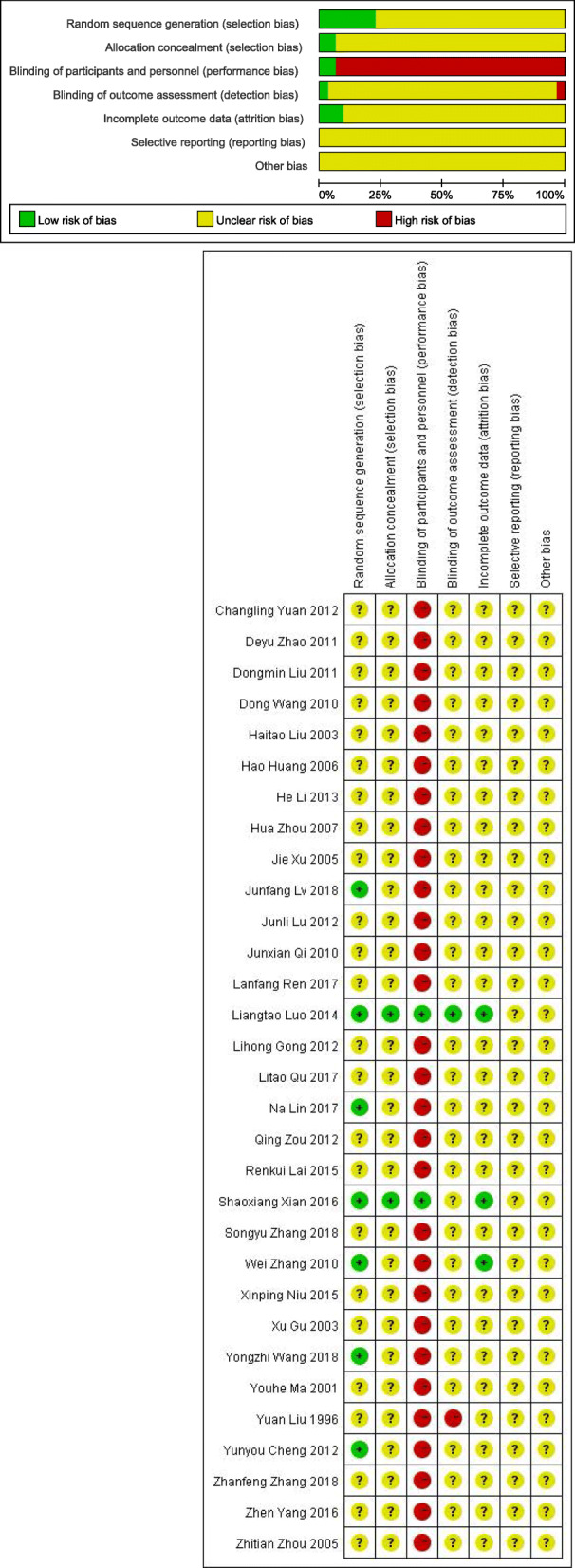
Risk of bias within studies

Among the 31 RCTs, only seven studies [21, 28, 30, 39, 40, 43, 44] used “random number table” or statistical software to generate the random sequence, the others just mentioned “random” but no description of specific methods. Two studies [39, 40] described allocation concealment and the blinding methods. Three studies [30, 39, 40] reported the case abscission and withdrawal. Generally, the risk of bias within the included RCTs was classified as high (See Fig. 2).

### Results of individual studies

#### Reporting of outcome measures

Outcome measures in the included RCTs differed. As the end points of CHF, mortality and rehospitalization were only reported by 4 studies (4/31, 12.90%), the other studies all reported surrogate outcomes, including efficacy of cardiac function (83.87%), left ventricular ejection fraction (LVEF)(54.84%), 6 min’ walk distance (6MWD)(45.16%) and brain natriuretic peptide (BNP)(16.13%). No studies reported related cardiovascular events. Seventeen studies (17/31, 54.84%) reported adverse drug reactions (ADRs), while 14 studies (14/31, 45.16%) did not report any safety measures.

**Fig. 3:**
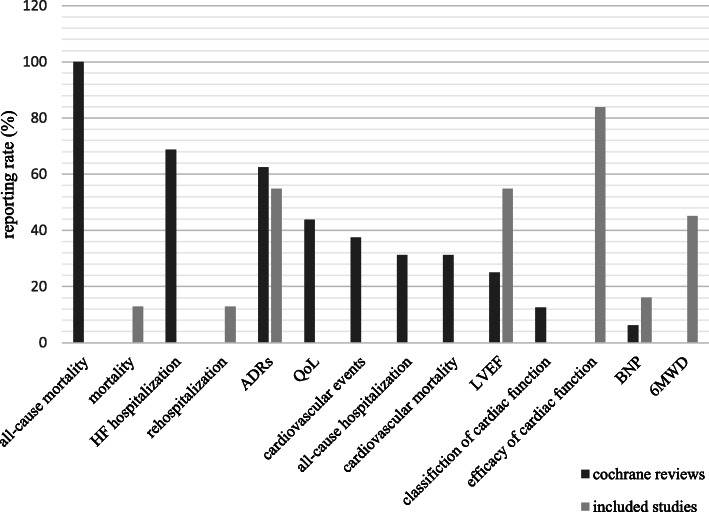
Outcomes reporting rate

By contrast, all of the CSRs of CHF reported all-cause mortality (16/16, 100%), focused on the end points and safety measures and analyzed the all-cause and specific-cause mortality or hospitalization respectively. The overall reporting of outcome measures is shown in Table 3 and Fig. 3.

**Table 3:** Overall reporting of outcome measures

Outcome measures	Included trials, n (%)^a^	Cochrane systematic reviews, n (%)^b^
All-cause mortality	0 (0)	16 (100)
Mortality	4 (12.90)	/
HF hospitalization	0 (0)	11 (68.75)
Rehospitalization	4 (12.90)	/
ADRs	17 (54.84)	10 (62.50)
QoL	0 (0)	7 (43.75)
Cardiovascular events	0 (0)	6 (37.50)
All-cause hospitalization	0 (0)	5 (31.25)
Cardiovascular mortality	0 (0)	5 (31.25)
LVEF	17 (54.84)	4 (25)
Classification of cardiac function	0 (0)	2 (12.50)
Efficacy of cardiac function	26 (83.87)	0 (0)
BNP	5 (16.13)	1 (6.25)
6MWD	14 (45.16)	0 (0)

Notes: *HF* Heart failure, *ADRs* Adverse drug reactions, *QoL* Quality of life, *LVEF* Left ventricular ejection fraction, *BNP* Brain natriuretic peptide, *6MWD* 6 min’ walk distance; ^a^reporting rate of included trials = n/31, ^b^of Cochrane systematic reviews = n/16

### Additional analysis

#### Reporting quality of outcome measures

All 31 RCTs reported the specific definition of outcomes, while only two [39, 40] clearly stated the primary and secondary outcome measures which were considered as high reporting quality of outcomes. Eight studies [14, 17, 29, 31, 32, 34, 35, 41] explained the use of the outcomes they had reported and five [19, 21, 23, 28, 40] adopted methods to enhance the quality of the outcome measurement, including training the investigators and arranging executives to measure the outcomes. Tables 4 and 5 shows the assessment of outcome reporting quality [13].

**Table 4:** Reporting status of each item for the assessment of outcome reporting quality

Study ID	Reporting quality of outcome measures [13]					
	1. Is the primary outcome clearly stated?	2. Is the primary outcome clearly defined so that another researcher would be able to reproduce its measurement?	3. Are the secondary outcomes clearly stated?	4. Are the secondary outcomes clearly defined?	5. Do the authors explain the use of the outcomes they have selected?	6. Are methods used to enhance the quality of outcome measurement if appropriate?
Junxian Qi 2010 [14]		x			x	
Dong Wang 2010 [15]		x				
Lanfang Ren 2017 [16]		x				
Zhitian Zhou 2005 [17]		x			x	
Changling Yuan 2012 [18]		x				
Litao Qu 2017 [19]		x				x
Qing Zou 2012 [20]		x				
Yunyou Cheng 2012 [21]		x				x
Lihong Gong 2012 [22]		x				
Junli Lu 2012 [23]		x				x
Xu Gu 2003 [24]		x				
Dongmin Liu 2011 [25]		x				
Hua Zhou 2007 [26]		x				
Renkui Lai 2015 [27]		x				
Na Lin 2017 [28]		x				x
Deyu Zhao 2011 [29]		x			x	
Wei Zhang 2010 [30]		x				
He Li 2013 [31]		x			x	
Hao Huang 2006 [32]		x			x	
Zhen Yang 2016 [33]		x				
Xinping Niu 2015 [34]		x			x	
Jie Xu 2005 [35]		x			x	
Haitao Liu 2003 [35]		x				
Youhe Ma 2001 [37]		x				
Yuan Liu 1996 [38]		x				
Shaoxiang Xian 2016 [39]	x	x	x	x		
Liangtao Luo 2014 [40]	x	x	x	x		x
Zhanfeng Zhang 2018 [41]		x			x	
Songyu Zhang 2018 [42]		x				
Yongzhi Wang 2018 [43]		x				
Junfang Lv 2018 [44]		x				

Notes: x in the column represents the study reported the corresponding item; empty columns indicate the study did not mention the item; studies with clear definitions of outcomes which did not preset primary or secondary outcomes were noted x in item 2

**Table 5:** Reporting rate of the items for assessment of outcome reporting quality

Items for assessment of outcome reporting quality	Reported trials, n (%)^a^
1. Is the primary outcome clearly stated?	2 (6.45)
2. Is the primary outcome clearly defined so that another researcher would be able to reproduce its measurement?	31 (100)
3. Are the secondary outcomes clearly stated?	2 (6.45)
4. Are the secondary outcomes clearly defined?	2 (6.45)
5. Do the authors explain the use of the outcomes they have selected?	8 (25.81)
6. Are methods used to enhance the quality of outcome measurement if appropriate?	5 (16.13)

Notes:^a^ reporting rate of each item = n/31

## Discussion

This systematic review mainly analyzed outcome measures in RCTs which assessed the efficacy of TCM in treating CHF caused by CHD. We included 31 trials meeting the eligibility criteria and extracted outcome measures from these studies. The outcome measures were mortality, rehospitalization, efficacy of cardiac function, LVEF, 6MWD and BNP, of which mortality and rehospitalization are end points for patients with CHF while the others are surrogate outcomes [61]. Only four studies (4/31, 12.90%) reported mortality or rehospitalization, and in comparison, all 16 CSRs of CHF analyzed all-cause mortality. This difference indicated that present TCM trials mostly assessed the surrogate outcomes and lacked evaluation of CHF end points.

In this review, nearly half of the included studies (14/31, 45.16%) did not mention any ADRs or adverse events, which apparently affected the safety assessment.

Apart from the problems of selecting outcome measures, the reporting quality of outcome measures was generally low, twenty-nine (93.55%) trials did not define the primary and secondary outcomes, which would confuse readers about major objectives of the trials and what the interventions really can improve.

In terms of methodology of the included RCTs, there were only two RCTs [39, 40] considered as high-quality. In general, the risk of bias of these trials was classified as high. We considered that the design and implementation of most studies were far away from an optimal RCT in random sequence, allocation concealment, blinding, statistics and reporting.

The selection of outcome measures is a critically important step in clinical trials. Scientifically rigorous outcomes could show significant and comprehensive information about the efficacy and safety of specific intervention [62], which would produce positive impact on clinical choices and decisions for physicians. In large-scale trials of heart failure, end points like mortality and hospitalization, were mostly set as primary outcomes [63, 64] and treatments that could reduce mortality or morbidity would be recommended in influential clinical guidelines [65, 66]. We did comparative analysis with CSRs, which are commonly agreed as high-quality information for making health decisions, to identify the present problems with outcome measures in studies conducted by TCM researchers. It was found that evaluation of improving clinical symptoms without robust evidence of clinical end points might be the primary reason why TCM interventions have not been widely recognized [67].

**Fig. 4:**
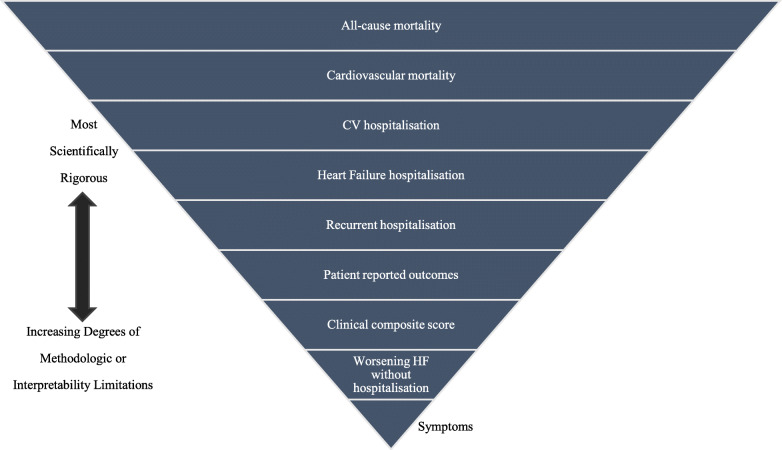
Schematic of outcomes for chronic heart failure trials [61]

A European Society of Cardiology (ESC) consensus on the outcomes of HF trials [61], which was included in the Core Outcome Measures in Effectiveness Trials (COMET) database, highlighted that clinical end points could support the consolidation of therapeutic strategies. Whilst surrogate outcomes reflecting manifestations are typically applied in earlier phases of drug or device development to support proof-of-concept (Fig. 4). We recommended that the future TCM trials could refer to this consensus to select outcome measures.

The assessment of safety is indispensable for any clinical trial. In the included RCTs, CHF patients secondary to CHD, mostly hade one or more comorbid conditions that would potentially cause treatment conflict [68]. Researchers should attach great importance to ADRs, adverse events or other safety outcomes throughout the studies and have the responsibility to estimate whether the intervention has a negative impact on patients or aggravates heart failure subsequently affecting mortality or hospitalization [69]. It is strongly recommended that TCM researchers should pay enough attention to the evaluation and reporting of safety in each trial.

Through this review, we proposed that TCM clinical trials should focus on the assessment of clinical endpoints when evaluating TCM interventions in treating CHF. Whereas, we were aware that the included trials were all too small to assess clinical endpoints. Whether the quantity of participants, the duration of the trial or the involved areas, these trials cannot be regarded as large-scale trials. The shortest duration of the included trials was 1 week [39] in which it seemed to be impossible to record mortality, rehospitalization or other endpoints. Actually, there might be difference of the endpoints between treatment and control group when the follow-up time was longer than or equal to 6 months in clinical trials [20, 22].

It is indeed difficult to conduct a TCM trial with certain size and duration to evaluate endpoints of heart failure, which would need appropriate organization and funding. We need high-quality prospective, multicenter RCTs [11, 70] rather than the present repetitive trials within a limited scale to promote the benign development of TCM [71]. We recommend collaboration among hospitals, research institutes and enterprises of TCM to conduct multicenter clinical trials to assess endpoints and generate convincing evidence which could guide the TCM clinical practice in a real sense.

This review has several limitations. First, Thirty-one trials might not be enough to analyze various outcome measures. Second, neither our review nor the included trials distinguished heart failure with reduced ejection fraction or preserved ejection fraction, which would affect the selection and evaluation of corresponding outcome measures. Third, the proportion and reporting quality of the outcomes we analyzed in the review cannot involve comprehensive information about outcome measures in RCTs. The methods to measure the outcomes, timing of measurement, how to enhance the quality of outcome measurement, follow-up of the primary outcomes and the assessment of composite outcomes are all significant factors discussing outcome measures and our future research will focus on these problems. Fourth, due to the aims of the review, we did not conduct meta-analysis within the 31 RCTs. In the future, we would include trials without or with low heterogeneity, comprehensively analyze outcomes and evaluate the efficacy and safety of TCM treatments.

## Conclusions

Several problems with the outcomes existed in present trials of TCM in treating CHF caused by CHD, including the lack of concentration on the clinical end points of HF, adequate safety evaluation, together with the low reporting quality. Moreover, the risk of bias was classified as high. In order to produce robust and convincing evidence for TCM in treating CHF caused by CHD, further studies should be rigorous and well-designed, set clinical end points as the primary outcome measures and strengthen evaluation of safety.

## Supplementary Information

**Additional file 1.** Search strategy.

**Additional file 2.** PRISMA checklist.

**Additional file 3.** list of excluded articles.

## Data Availability

All data and materials analyzed supporting the conclusions of this article are included within the article and the additional files.

## Abbreviations

6MWD: 6 min’ walk distance
ADRs: Adverse drug reactions
BNP: Brain natriuretic peptide
CHD: Coronary heart disease
CHF: Chronic heart failure
CSR: Cochrane systematic review
HF: Heart failure
LVEF: Left ventricular ejection fraction
QoL: Quality of life
RCT: Randomized controlled trial
TCM: Traditional Chinese medicine
