# Supplementary material for: Problems with the outcome measures in randomized controlled trials of traditional Chinese medicine in treating chronic heart failure caused by coronary heart disease: a systematic review
Source: BMC Complement Med Ther. 2021 Aug 31;21:217. doi: 10.1186/s12906-021-03378-z (PMC8406575; doi:10.1186/s12906-021-03378-z)
Supplement: Supplementary file 1 — Additional file 1. Search strategy. [file 12906_2021_3378_MOESM1_ESM.pdf]

Additional file 1: search strategy

#1 Heart failure [MeSH Terms]

#2 (Heart failure [Title/Abstract] OR Cardiac Failure [Title/Abstract] OR Heart Decompensation[Title/Abstract] OR Myocardial Failure[Title/Abstract] OR cardiac decompensation[Title/Abstract] OR cardiac incompetence[Title/Abstract] OR cardiac insufficiency[Title/Abstract] OR heart insufficiency[Title/Abstract] OR HF[Title/Abstract])

#3 #1 OR #2

#4 Medicine, Chinese Traditional [MeSH Terms]

#5 (traditional Chinese medicine [Title/Abstract] OR TCM [Title/Abstract] OR Chinese medical treatment[Title/Abstract])

#6 #4 OR #5

#7 Randomized Controlled Trial [Publication Type]

#8 (randomized controlled trial [Title/Abstract] OR random*[Title/Abstract] OR control* study [Title/Abstract] OR control* trial[Title/Abstract])

#9 #7 OR #8

#10 #3 AND #6 AND #9
