# Supplementary material for: Problems with the outcome measures in randomized controlled trials of traditional Chinese medicine in treating chronic heart failure caused by coronary heart disease: a systematic review
Source: BMC Complement Med Ther. 2021 Aug 31;21:217. doi: 10.1186/s12906-021-03378-z (PMC8406575; doi:10.1186/s12906-021-03378-z)
Supplement: Supplementary file 3 — Additional file 3. list of excluded articles. [file 12906_2021_3378_MOESM3_ESM.pdf]

5 studies with error data

Author Year Title Volume Issue Pages

Xiaofei Li, et al. 2016 Clinical study on injection of Yiqi for the treatment of 39 cases of acute myocardial infarction complicated with left heart failure 38 2 262-264,271

Qin Song, et al. 2002 Clinical observation of 48 cases of acute myocardial infarction complicated with heart failure treated by Shenfu Injection 1

Qiang Liu, et al. 2009 Effect of Shenqi Huoxue Li Shui Recipe on brain natriuretic peptide and 6-minute Walking test in patients with chronic heart failure due to coronary heart disease 7

Gangjun Zong et al. 2007 Treatment of 34 cases of chronic heart failure with coronary heart disease by Nuodicang Capsule 26 5 513-514

Xiulian Gu et al. 2007 The curative effect of Musk Baoxin Pill on rehabilitation of patients with coronary heart disease and heart failure 16 1 92-94

Non-RCT

Author Year Title Volume Issue Pages

Shuai Wang, et al. 2013 Influencing factors and countermeasures of subject compliance in multi-center clinical study -- Based on the implementation of Qishen Yiqi dropping Pill in the clinical evaluation of coronary heart disease and heart failure 5 2 104-106

Meiqin Wei 2007 Clinical observation of Fuxin Decoction in the treatment of coronary heart disease diastolic heart failure5 12 1170-1171

Yuan Bian, et al. 2017 Progress in the main evaluation model of acute heart failure 04 396-403

Huan Tong 2017 The correlation between the prognosis of heart failure and blood lipid level in elderly patients with coronary heart disease 08 971-973

Weihua Xu 2016 Application of Xinbi Decoction in treating chronic heart failure of coronary heart disease with Qi deficiency and Blood Stasis syndrome 17

Xianming Liu, et al. 2006 Clinical observation of 30 cases of Coronary heart failure treated by Yiqi huoxue Fang 15 9 946-947

Cunfu Li 2012 Treatment of 25 cases of chronic congestive heart failure with integrated Chinese and Western medicine 32 9 1196-1197

Feixiang Fei 2013 Discussion on the treatment of chronic heart failure by Chinese medicine 11 1 90-91

Wenju Liu, et al. 2007 Qiliqiangxin capsule on ischemic cardiomyopathy patients with exercise tolerance and quality of life 13 8 885-887

Yiling Pu, et al. 2013 Yuan's Yangxin Oral liquid was used to treat 78 cases of chronic systolic heart failure with coronary heart disease 03 8-10

Not HF caused by CHD

Author Year Title Volume Issue Pages

Chaoguang Wang 2011 Effect of "Qiangxin Mixture" on plasma brain natriuretic peptide in patients with chronic heart failure 3 22 61-62

Yongqi Deng, et al. 1997 "Tongmai Qiangxin Yin" was used to treat 38 cases of chronic congestive heart failure 7

Shouhong Chen, et al. 2011 Effect of "Yixin cream" on cardiac function in patients with chronic congestive heart failure 43 2 21-22

Xinbin He, et al. 2010 6min walking test and heart color doppler ultrasonography evaluation of yangxin tongmai decoction in the treatment of chronic heart failure 8 9 1027-1029

Jian Lv 2015 Observation on the effect of warming Yang and activating blood circulation in 90 patients with chronic heart failure 27 7 122-123

Yamei Shen, et al. 2006 B ultrasonic observation of shengmai capsule on left ventricular remodeling and cardiac function in patients with congestive heart failure 29 2 70-71

Ao Jiang, et al. 2017 Clinical observation of CRRT combined with Traditional Chinese medicine in the treatment of intractable heart failure 15 11 162-163

Yuxin Su 2006 Baoxin Kangshuai Decoction was used to treat 41 cases of chronic congestive heart failure 27 2 132-133

Qiu Zhang, et al. 2016 Clinical observation of Baoyuan Decoction and Zhenwu Decoction combined with conventional Western medicine in the treatment of chronic heart failure 31 16 2406-2408

Xianwei Lu 2006 Baoyuan Decoction treated 30 cases of chronic congestive heart failure 27 7 772-773

Zhenrui Li2013 60 cases of chronic heart failure were treated with Bufei Yishenfang combined with Western medicine 33 10 1666-1667

Jie Li, et al. 2011 Clinical study on the treatment of chronic heart failure by invigorating kidney and activating blood circulation 9 12 1426-1427

Xiaohong Wang, et al. 2007 Effect of tonifying kidney and strengthening heart on chronic heart failure and exercise tolerance 5 6 529-530

Deling Kong, et al. 2014 Clinical observation on 51 cases of heart failure with Yang-deficient water type treated by Buxin Decoction 36 4 509-511

Jun Ren 2011 Clinical study of Buyang Huanwu Decoction in the treatment of chronic congestive heart failure 15 31 1052-1053

Meisong Liu, et al. 2014 Clinical efficacy evaluation of Buyiqiangxin Tablet in the treatment of congestive heart failure 4 16 19-22

Bo Chen, et al. 2005 Clinical study of Buyiqiangxin Tablet in the treatment of chronic heart failure 3 1 43-45

Liu Yuanlin;Zou Haidong;Huang Shixiang;Jay huang;CenZhao;Mr Bright;Gui-e Chen;Zhang Qiongdan 2007 Clinical efficacy and safety of Shengmai injection at different doses in the treatment of heart failure 20 8 35-37

David ku.YanJun;Wang Qiaozhong, 2005, analysis of the influence on cardiac function of 55 patients with heart failure. 5

Ke-chun Yin;Shang Xiangjiang;Zhou Wenbin 2004 Shenfuqi Injection in the treatment of congestive heart failure and its effect on oxygen free radical 11
Yin Kechun 2007 Shenfuqi Injection in the treatment of congestive heart failure and its effect on free radicals 1
Observation of therapeutic effect of Shenfu Shuxin Decoction on senile diastolic heart failure 11 25 119
Effect of Tian Jun 2003 Shenfu Injection on hemorheology in patients with congestive heart failure and its curative effect observation 6
Xu-peng jin;Guo Jingshu 2007 Effects of Shenfu Injection on endothelial function in patients with congestive heart failure 16 4424-425
James tien.Effect of Shenfu injection on congestive heart failure 9
Li hui.Liu township;Observation on therapeutic Effect of Shenfu Injection on congestive heart failure 4
Zhao Ling;Therapeutic Effect of Shenfu Injection on congestive heart failure 33 33 9
Wendy lee;Yao Rui;Li li;Chen;Zhao Guojun;Hao Zhengyang;Feng Shengdong;Kong Lingyao;A randomized controlled clinical study of Zhang Yanzhou's shenfu Injection in the treatment of acute decompensated heart failure
YanKaiYun; Ren Huifeng 2011 Shenfu Injection in treatment of 36 cases of acute left heart failure 32 10 1280-1281
Jia qiao; Observation and nursing of Senile chronic heart failure treated by Shenfu Injection 7
Song Shengqing; Hong-hui cheng;Huang Pingdong;Huang Junhui;LingQinLiang;Effect of Shenfu Injection on chronic systolic heart failure 24, 8, 42-43
Dong Guiqin;Lin Nan 2005 Shenfu injection in the treatment of 100 cases of heart failure 1
Fan Lihua;Xiao-bing li;Li Qinghai 2009 Shenmai Ningxin Mixture in the treatment of 40 cases of chronic heart failure clinical observation 5
Ye Xiaohan;NingWeiMin;He Shidong 2005 Shenmai syrup in treating 72 cases of chronic cardiac insufficiency 3
Wang;Hong-ling hu;Effect of Qianduan 2004 Shenmai Injection on hemodynamics in patients with congestive heart failure 5
Pan Zheng;Pershing longevity;Huang Xiannan 2006 Influence of Shenmai Injection on heart rate variability in patients with chronic severe heart failure 34 6,600-601
Cao Fengzhen;Clinical observation of 30 cases of congestive heart failure treated by Shenmai Injection 2007
Yin Fuqiang 2006 Shenmai injection in the treatment of 50 cases of congestive heart failure
Qu Fan 2006 Shenmai Injection in the treatment of congestive heart failure clinical observation 15 10 1102-1103
Zhang wei.Clinical observation of Shenmai Injection in the treatment of senile Congestive heart failure 9
Gao Jing 2005 Shenmai injection in the treatment of 30 cases of chronic congestive heart failure 3
ZhaiSuiYan;Cai Xiaolu;Wen Shaojin 2006 Shenmai Injection in the treatment of 48 cases of chronic congestive heart failure
Qu Tangqing 2003 Shenmai injection in the treatment of 60 cases of chronic heart failure clinical observation 4
Xiao-ling Yang;What ShiMin;Effect of Shenmai injection on chronic heart failure 27, 4, 16-17

NiHaiTao;MAO Jianhui 2006 Shenmai injection in the treatment of 84 cases of heart failure 4 10 902-903
Wen-ming Yang;Zhou Yixuan 1997 Therapeutic effect of Shenmai injection on heart failure 7
Ching-hwa Yang;South hong mei;Christy.Wei-chun leng.Liu Yufu 1997 Clinical observation of Shenmai injection in the treatment of left cardiac insufficiency 4
Gu Huimin 2006 Shenmai injection in the treatment of 30 cases of chronic congestive heart failure clinical observation 21, 4, 40-41
AnYanRong;Wei;Effect of 2006 Shenqi Fuzheng Injection on brain natriuretic peptide concentration in patients with heart failure 28 6 475-476
Section of the building;Guo Chuan 2007 Shenqi Fuzheng Injection and Shuangba Mixture in the treatment of chronic heart failure clinical observation 9, 4, 54
Had less established;Clinical observation of Shenqi Fuzheng Injection in the treatment of congestive heart failure 9
Tong Qiang 2005 Shenqi Fuzheng Injection in the treatment of refractory heart failure Clinical observation 4
A xing wei;Xing-hua li;Clinical observation of 128 cases of heart failure treated by Shenqi Fuzheng Injection 2006
YuZhengKe;Jiang-bo huang;Zhu ;Hu Lian;Chen Zhihong 2001 Shenqi Granule in the treatment of 98 cases of chronic congestive heart failure
Tzu-ming chang;Xiang-hong Yang;Yu-qin song;Zhang Lihua;MiaoJie;Li Yuming;Tian-tao zhang;Red;Clinical study on the treatment of Heart failure by Shenqi Zhenwu Granule 28 12 895-896
Liu Yingsheng;Fix the plaintext;FuJinXiang;De-quan liu;Influence of 2011 Shensong Yangxin Capsule on rehospitalization rate of patients with chronic heart failure 11 22 5330-5331
Sun yuan ying; Mao-sung kuo, et al. 2006 Therapeutic effect observation on 42 cases of refractory heart failure treated with Shenwu Guanxin Granule 40 4 16-17
YuZhengKe;Akaka;Xiao-jing zhu;Zhi-hong Chen;Zhu edges;Tan Yujun 2013 Clinical Study on the effect of Shenzhu Xinkang Decoction on the quality of life of patients with chronic heart failure 09 9-12
Huang wq.Rong-de tang;Zhong Rongguang;Zhao Baiqing;Zhen-rong Lin;Bird.He Hongbing;Guan Hailin 2013 Clinical study of Chen Fu Qiangxin Decoction in the treatment of chronic heart failure with phlegm 11, 7781-783
Mary;Yang Lan;Chen Tianduo;Li Peijie 2003 effect of large dose shengmai injection on coagulation function in patients with chronic heart failure 4
Li Guohua 2012 Dan Hong Bushen Decoction for the treatment of chronic congestive heart failure curative effect observation 27 5 888-889
Jia Haiping 2011 Danhong Injection for treating 42 cases of chronic congestive heart failure 32 6 660-661
Li ping.
Summer MeiHua;Seats in early spring;Hao Shukun;Shi Hongxing 2015 Danqi Yangxin Granule in the treatment of 49 cases of chronic heart failure clinical observation 37 2 183-185
Zou Xu;Guang-ming pan;Ze-yin liu;Lin Xiaozhong;Observation on the efficacy of Deng Tietao Nuxin Capsule in treating heart failure with qi deficiency and blood stasis

YanXia;Wen Wang show;Wu Huan-Lin 1999 Effect of Diao Huangqi injection on chronic heart failure 5
Wen-bin zhou;He Huiming;NieWenJuan; Clinical Observation of Fuzheng Guben Wenyang Tongmai Cream in the treatment of Chronic heart failure with Yang Deficiency and Blood stasis: Observation on the therapeutic effect of 2013 Poria Sini Decoction on chronic heart failure 45 8 14-15
Cao Hang;Summary of 30 cases of congestive heart failure treated with Fuzi Qiangxin Capsule
Fang Xueying;Hu Ruifu 2005 compound Salvia miltiorrhiza dropping Pill for 65 cases of senile heart failure 6
Weng Huiyuan 2002 Electrocardiogram analysis of compound Danshen dropping pills before and after treatment of cardiac insufficiency 4
Chen Xunshan;Yu Tiantai 2017 Fuyuan Decoction for the Treatment of heart failure syndrome of Heart and Kidney Yang Deficiency 02 151-153
Chen Xunshan;Observation on the curative Effect of Yu Tiantai 2017 Fuyuan Decoction in treating the syndrome of Xinzhong Disease, Xinshenyangxu syndrome 35 2 151-153
Analysis on the short-term efficacy of puerarin injection in the treatment of chronic congestive heart failure 17, 10, 27,155
Mr Xu.Yu wen;Li Qiuhua;Yang Zhongqi, 2012: A clinical study on the treatment of chronic heart failure by drinking by drinking 4 1 3-4
Wu;Zhang Shanshan 2015 Guben Zhuyang Prescription for the treatment of chronic heart failure with Heart and kidney Yang Deficiency 12
Jevons lee;Emily lau.Effect of Guizhi Decoction on left ventricular function in patients with congestive heart failure 1
Song Fangli;Zhong Xu;Wang Luzhuo 2014 Guizhi Yangxin Prescription adjuvant treatment of chronic heart failure clinical observation 33 9 9
Xiao-li jing;Dong-ping wong.Liu Jianbo 2006 Huangqi Shenmai Wuling Decoction for the treatment of congestive heart failure clinical study 15 1 8-10
Effect of 2007 Huangqi Shengmai Decoction on 6-minute walking test in patients with chronic heart failure 23, 17
Therapeutic Effect of Huangqi Zhenwu Decoction and Wuling Powder on chronic heart failure 10
What from.Li Li 2007 Huangqi Zhenwu Tang in the treatment of chronic congestive heart failure syndrome of Heart and kidney Yang Deficiency clinical observation 27 2 62-63
Wei yulin;Chu-qiang li;Chen Xilong;Liu Yingmei;ROM. Mulberry 2006 astragalus injection cytokines in patients with congestive heart failure and the effect of angiotensin Ⅱ 10 3 54-56
Duan Ying 2006 Astragalus Injection on the effect of left ventricular remodeling in congestive heart failure clinical study 29 3 19-20
Zhang jl.Lian-wang jia;Effect of Astragalus injection on CHF cardiac function and hemorheology in the elderly 4
Jin-guo zhang;Tom kao.Hong-yong tan; et al. Effect of Astragalus injection 2006 on plasma pro-inflammatory cytokines in elderly congestive heart failure patients 29 2 13-15
Su Jingze;Effect of Tan Hong 2017 Astragalus Injection on quality of life and N-terminal brain natriuretic peptide Precursor in patients with chronic heart failure 07

678-680
Jin-guo zhang;Monakhov;He;Wei Guang, et al. Effect of 2005 Astragalus injection on plasma apoptotic factors in patients with chronic heart failure 5
Cordierite;Wu Xianwang;Zhang Jingfang 2005 Astragalus injection in treating 61 cases of congestive heart failure observation 3
Zhou Zhilin;Yu ping.Lin Ding;Liang Lizhen;Effect of 2001 Astragalus injection on congestive heart failure 10
Room ze adai;Qin Yuanling 2003 Astragalus injection in the treatment of congestive heart failure clinical observation 14
Xue Zhenhuai;Huang Bo ladder;Clinical observation of 2007 Astragalus injection in the treatment of congestive heart failure 26 5 10-11
Liu Yingqin;Li Yun 2005 Astragalus Injection in the treatment of congestive heart failure clinical study 4
Wang;Gao Sheng 2002 Astragalus Injection in the treatment of senile Congestive heart failure 6
Clinical efficacy of Yuanguangping 2003 Astragalus injection in the treatment of chronic heart failure 6
Feng Chunlin 2008 Astragalus injection in the treatment of 66 cases of chronic heart failure observation 01 78
Wang;Zhou Xuelin 2010 Huiyang Jixin Decoction for the treatment of 82 cases of chronic heart failure: 30, 4, 374-375
Chai Hongjia;Clinical Observation of Yan Xia 2016 Huoxue Qiangxin Prescription in the treatment of chronic congestive Heart failure 7
Guo-hong zhao;Li Ping 2009 Therapeutic Effect of Huoxue Shengmai Powder on chronic heart failure 9
Liu Zhen;Observation on the therapeutic effect of Jisheng Shenqi Pill on diastolic heart failure with deficiency of heart and kidney Yang
Wang Huisi;Zhang Xusheng;Zhang Manzhi 2005 Clinical study on the treatment of congestive heart failure by Adding and reducing Wood Fangji Decoction 1
Huang Jun ao.Zhao Xiaoqin 2015 Clinical Study on the treatment of Chronic cardiac insufficiency with Additive and Subtractive Zhenwu Decoction 7 23 64-65
Chen Kui;Dong-sheng hu;Lu Bingfeng 2002 Effect of Jiawei Buyang Huanwu Decoction on cardiac function of senile heart failure patients 11
Li hui.Li ah;Liu township;Yang Wei;Liu Xiaojun 2005 Treatment of congestive heart failure and its effect on thyroid hormone 2
Zhang Zhixiang;Ma Hong-su 2006 Jiawei Linggui Zhugan Decoction for the treatment of 36 cases of chronic heart failure 27 7 771-772
Pan Guangqiang;Hu Yongqi;Ye Xiangyang 2005 Clinical study of Jiawei Shengmai Decoction in the treatment of congestive heart failure
Clinical observation of 50 cases of chronic heart failure treated by Jiawei Zhenwu Decoction 21, 7, 34-36
Yu Qingbiao;Liu Zhong added Shenfu injection to treat 81 cases of refractory heart failure in 1997
Xiao-fen ruan.Mei-xian jiang;Xu Yan;Cui Song;Adan.
Effects of Kanli Decoction on activity tolerance, quality of life and aggravation frequency of heart failure in patients with chronic heart failure
Li Jie 2011 Effect of Jianpi Huoxue Decoction on cardiac function of patients with chronic congestive heart failure 9, 767-768
Li Xinyue;GanHaoYun;Clinical Observation of Decoction for Invigorating Spleen and Nourishing Heart in rehabilitation treatment of chronic heart failure 3

Hai-bin zhao;Shen Chengling;Liu Jinmin 2004 Effect of Jianxin Decoction on serum cytokines in patients with congestive heart failure 3

Hai-bin zhao;Shen Chengling;Liu Jinmin 2004 Effect of Jianxin Decoction on serum cytokines in patients with congestive heart failure 03 186-187+194+5

Kang Genchao;Wen-ke zhang;Wang Yunqiang;Zhang Junsheng 2011 Jiuwei Qiangxin Decoction for the treatment of 60 cases of chronic heart failure

Kang Genchao;Wen-ke zhang;Wang Yunqiang;Zhang Junsheng 2011 Jiuwei Qiangxin Decoction for the treatment of 60 cases of chronic heart failure 07/814

Zhang jie.Yu Xiwen;Wu Ningbo 2011 Carvedilol combined with Qishen Yiqi dropping Pills in the treatment of 40 cases of chronic congestive heart failure clinical observation 24, 608-610

Yun-zhi Chen;Yao-yao wang;Lv Jianwei;Guo;Effect of 2007 Kangxin Decoction on Testosterone in patients with congestive heart failure

Li-xin hu;Zhang Zhanying;Yan-ling li;Effects of Yunyun 2007 Kangsenbaoxin Decoction on plasma nitric oxide and endothelin in congestive heart failure

Do won chang; De-quan liu; Yang Shu-lian 2006 Anti-Heart failure mixture treatment of 46 cases of refractory heart failure clinical observation 15 9 948-949

Study on the application value of Zhenwuqiangxin Decoction in the treatment of senile Heart failure

Li Aimin 2006 Lishen Injection in the treatment of congestive heart failure clinical observation 15 6 570,578

Liru Wang. Clinical observation of 42 cases of congestive heart failure treated with Lishui Qiangxin Decoction combined with western medicine 30 12 1296-1297

Wang Zhanzhan;Bird wave;Clinical Observation on the treatment of chronic heart failure with diuretic resistance by Lishui Decoction 10, 24, 87-88

Wang Baoshen, et al. 2006 Ling GUI Sanshen Decoction for the treatment of chronic congestive heart failure curative effect observation 15 7 689,691

Li Xiaoqiu;GengXiaoYin;Sha-yan wang;Hong-yu jiang;Clinical study on The treatment of congestive heart failure by Linggui Zhugan Decoction (2005) 4

Zhou Hua 2006 Effect of Antler prescription on left ventricular remodeling in patients with chronic heart failure 40 4 13-15

Cai Hui;Hu Wanying;Yan-jun wang;Zhang Junhui;Guo Jun hao;Observation of curative effect of Lujiao Prescription on congestive heart failure 4

Ding-you Yang, et al. 2003 Clinical study on the treatment of Congestive heart failure with Xinshenyang-deficiency Oral Liquid 11

Lin Xiaozhong, et al. 2011 Clinical study of "Yin-yang divide treatment" comprehensive treatment plan for chronic heart failure 43 3 16-18

Lin Xiaozhong, et al. 2011 Clinical Study on the "Yin-yang divided Treatment" comprehensive Treatment Plan for chronic heart failure 03 16-18

Sun Xiaoping;Observation of traditional Chinese medicine clinical efficacy of chronic heart failure 14, 2, 187

Zou Xu;Ze-yin liu;Guang-ming pan;Influence of Maishu Peach 2006 Nuxin Capsule on 6-minute walking Distance in patients with chronic heart failure 33 8 915-916

MaiShu Tao, et al. 2008 Warm heart Capsule on non-invasive hemodynamics in patients with chronic heart failure 35 6 808-809

Ze-yin Liu, et al. Clinical study on the effects of Maishu Peach 2007 Nuxin Capsule on ventricular diastolic function in patients with diastolic heart failure 39 7 21-22

Zou Xu, et al. Long-term effect of Luo Ying 2006 Nuxin Capsule on chronic congestive heart failure 40 2 6-7

Zhang Zhihui;Yuan Yanzhen 2013 Qishen Yiqi Dropping Pill effect on heart function and Lymphocyte subgroups in elderly patients with heart failure 10 31 26-28

Jun-ling zhang;Effect of qishen Yiqi dropping Pills 2013 on plasma natriuretic peptide and cardiac function in patients with diastolic heart failure 29 1 132-134

Effect of Yubo 2012 Qishen Yiqi dropping Pill in the treatment of senile chronic congestive heart failure: 21 8 383-385

Mengmoke 2010 Qishen Yiqi dropping Pills in the treatment of elderly chronic heart failure effect observation 10 13 3097-3098

Effect of qishen Yiqi dropping Pill on 74 cases of chronic congestive heart failure 5 2 135-137

Forestry;Pan Wenjing;Bao Yongjian clinical Observation of 2010 Qishen Yiqi dropping Pill in treating chronic congestive heart failure 10

He Liuping;Wu Yufu 2009 Qishen Yiqi Dropping Pills in the treatment of chronic congestive heart failure sinus Rhythm concussion Clinical study 26 3 202-204

Xu Jing 2017 Qishen Yiqi dropping Pills in the treatment of 146 cases of chronic heart failure observation 29 2 189-190

Clinical Observation of Shao Xuesong 2015 Qishen Yiqi dropping Pill in treating chronic heart failure 15

Analysis on the clinical effect of 2016 Qishen Yiqi dropping Pill in treating chronic heart failure 6, 15, 62-64,207

Liu;Observation on curative effect of Qishen Yiqi dropping Pill in treating chronic heart failure 19 6 112-113

Liao Yuxiu 2008 Qishen Yiqi dropping Pill for the treatment of chronic heart failure effect Observation 17 25 3928-3929

Chen Hui clinical Observation of 2016 Qishen Yiqi Dropping Pill in the treatment of heart failure with reduced ejection fraction of Qi deficiency and blood stasis type

Luo Jianhua 2007 Qishen Yiqi dropping Pills in the treatment of asymptomatic heart failure observation 1, 4, 68-69

Wen-jun cheng;XiJianJun;Clinical Observation of 2016 Qiliqiangxin Capsule in the treatment of elderly patients with chronic heart failure 38 6 927-929

Kangling 2009 Qiliqiangxin capsule treatment of 30 cases of chronic heart failure clinical observation 7 12 1466-1467

Hui-fen Wang, et al. 2011 Qiliqiangxin capsule treatment of chronic heart failure 54 cases summary 27 5 1-2

He Shaolei 2016 Qiliqiangxin capsule treatment of chronic heart failure 80 cases of clinical efficacy 32 11 1707-1708

Effect observation of qiliqiangxin Capsule in treating chronic heart failure 31, 665-666,669

Wang Xiuzhen 2012 Qiliqiangxin capsule treatment of chronic heart failure curative effect observation 27 8 1566-1567

Shan Wu, et al. Xuedong 2007 Qiliqiangxin capsule in the treatment of chronic heart failure randomized double-blind, multi-center clinical study 6, 263-266

Effect analysis of 26 cases of congestive heart failure treated with Qiliqiangxin Decoction.

Wang Zhenkui;Du Minhua;Liu Ning 2011 Qi Maiyixin Granule in the treatment of heart failure clinical observation 3 8 24-25

Wang Bao and;Sun Lanjun;Clinical observation of 134 cases of congestive heart failure treated with Jiangxin Granule in 1997 4

Ye Shuhui 2005 Effect of Qiangxin Mixture on left ventricular function in 48 patients with chronic congestive heart failure 2

Guo-hui zou.Zhong-yong liu;Zhang li;Effect of Qiangxin Mixture on plasma brain natriuretic peptide in patients with congestive heart failure.

Xiao-hu Chen;Tang shu hua;As rev to;Clinical observation of 30 cases of congestive heart failure treated by Decoction Fanying 1993 Qiangxin Mixture 0 1 15-17

Liu Yan 2007 Qiangxin Mixture treatment of congestive heart failure clinical observation 30 2 131-132
Li Xiao 2014 Qiangxin Capsule for the treatment of 30 senile chronic heart failure with mutual knot of Yangdeficient water and blood stasis
Chen Quanfu;Effect of Chen Xiaohu 2005 Qiangxin Granules on cytokines in patients with chronic heart failure 2
Xiao-ming he;XiaoJunQing;In your flat;An Hongze 2011 Qiangxinli Decoction for the treatment of 56 cases of chronic heart failure 20 12 80
Wang Yakuan;Ding Zhixin;Bai Xiaoyan, 2014 Clinical Study of Qiangxinningshuai Oral Liquid in the treatment of chronic Congestive Heart Failure 04 682-683
Yin Yuping;Yin Wenyin 2012 Qiangxin Decoction combined with Western medicine in the treatment of 26 cases of congestive heart failure
Li Sanmei 2003 Qiangxin Decoction for treating 36 cases of heart failure 5
Zhang Yan;Xu Zhuo;Bell music;Li Jingshu 2009 Clinical study of Qiangxintongmai Granule in the treatment of chronic heart failure 23 1 3-4
Zhao Hui;Liu;Sun Hong;Clinical Observation of Quphlegm Tongyang Decoction in treating chronic heart failure 28, 4, 109-110
Clinical observation of trimetazidine combined with Yangxinshi in the treatment of chronic heart failure 4
Li gang.To cui zhu;Clinical observation on the treatment of chronic heart failure with Bao Tingyu 2003
Kong Bin;Clinical observation of Ginseng and Wolfberry Decoction in the treatment of heart failure 3
Xiao-hua dai.Yi-xuan zhou;Yu Xingqun 1997 Ginseng injection in the treatment of 69 cases of congestive heart failure 5
Hughs;Cheng Xiaowei;Zhang Yulei 2007 Musk Heart-protecting Pill in the treatment of chronic heart failure value 34 7 80-81
Liu Shuyun 2006 Clinical study of Shengmai Astragalus injection in the treatment of chronic congestive heart failure in the elderly 38 10 926-927
RuiGuoHua;Zhen-yi liu;Treatment of 35 cases of chronic congestive heart failure with Shengmai Activating Heart Decoction
Li ke-zhong 2009 shengmai qiangxin tang in the treatment of 40 cases of chronic heart failure: 25, 88,116
Xiao-mei hu;Effect of Han Zhenhua 2004 Shengmai Injection on patients with congestive heart failure 10
Analysis of the effect of Yangyongyi 2001 Shengmai Injection on senile chronic cardiac insufficiency 9
Jing-yuan MAO, et al. 2003 Shengmai Injection on blood concentration and pharmacokinetic parameters of Digoxin in heart failure patients.
Shi Zhibo;Zhang Yanyun;Observation on the curative effect of Shengmai injection in the treatment of chronic congestive heart failure
Yang Shunhong, et al. 2006 Shengmai Injection for treatment of congestive heart failure 4 10 913-914
Jiang Shuqin 2006 Shengmai Injection in the treatment of congestive heart failure effect observation 15 3 264
Wei-jian xu;Huang Yi;Clinical observation of Qu Songbai 2003 Shengmai Injection in treating congestive heart failure 5
Jionglong shao, et al. 2006 Shengmai Injection in the treatment of senile chronic heart failure 8, 4216-217
Ding Qizhu 2005 Clinical observation of Shengmai Injection in the treatment of cardiac insufficiency 5

Mei-xian Jiang, et al. 2000 Acoustic quantitative monitoring of cardiac function to evaluate the efficacy of Traditional Chinese medicine in the treatment of congestive heart failure 9

TengYuLian;Lu Xiaoyun 2003 Shuxinhe Prescription for the treatment of diastolic dysfunction in 60 cases of heart failure clinical observation 2

Jin lei check;Zhao Mingjun; Clinical observation of Wenyining 2011 Shuxin Decoction in treating 30 cases of chronic congestive heart failure

Liu Zhangxu 2014 To explore the application of Chinese medicine Wenyang Lishui method in the treatment of heart failure 23

Li Guiman. Observation on the therapeutic effect of left ventricular diastolic dysfunction heart failure by regulating qi and removing blood stasis and removing turbid

Wang Ma, et al. 2017 Harmonic Yingwei Method on cardiac autonomic nervous dysfunction in chronic heart failure 06 1380-1382

Liao Yihua;Deng Yunmei;Especially liuxiang.Deng retreat;Li Xiaoyi 2003 Clinical observation of Tongxinluo capsule in treating chronic heart failure 1

Yan-li Wang, et al. Prescription combined with Western medicine on plasma brain natriuretic peptide and 6-minute Walking test in patients with chronic congestive heart failure

Wang Jing, 2014 Tongyang Huoxue Prescription for the treatment of 40 cases of chronic congestive heart failure

Song Jing, et al. 2013 Effect of Wenbuxinshen Prescription on cardiac function and endostatin and angiotensin II in patients with chronic heart failure 25 3 16-18

Shi Yong, et al. 2017 Improvement of activity tolerance of Wenshen Huoxue Decoction in elderly patients with chronic heart failure with Yang Deficiency and blood stasis syndrome 23, 9, 184-189

Effect of Jinbao 2013 Wenshenyangxin Prescription on serum NO and JAK-1 levels in patients with chronic heart failure

Xie Qianglong, et al. 2008 Wen Shen Yi Xin Dan mainly treated 20 cases of congestive heart failure 23 11 1686-1688

Huai-fang yao, et al. 2005 Clinical observation on the treatment of congestive heart failure mainly with Wenshen Yixindan 4

Ye-xiang Zhang, et al. 2008 Wen Shen Yi Xin Dan clinical efficacy in the treatment of congestive heart failure and its influence on BNP level 17 2 137-139

Hou Wangang, et al. 2011 Clinical comparison of wentong method and Wentyang method in treating chronic congestive heart failure 30 10 697-698

Yu jia xin;Li Jianqiang 2016 Warming Yang kidney tonifying method combined with Bisoprolol in the treatment of 31 cases of chronic heart failure

2010 WenYangFang in patients with chronic congestive heart failure of plasma brain natriuretic factor, myocardial troponin Ⅰ level and the influence of 6 mins' walking test 8

Shang Qi, et al. 2012 Study on the Effect of Wenyang Prescription on the neuroendocrine of heart failure syndrome with Xinyang Deficiency 14 9 175-177

Clinical Observation on improvement of cardiac function and neuroendocrine indexes in patients with Heart failure and Heart Yang deficiency by Wenyang Prescription

Ye Ye;Clinical Observation of Wenyang Prescription in improving cardiac function and neuroendocrine indexes in patients with heart failure and Yang-deficiency 21 5 272-275

Shang Qi, et al. clinical Study on Wenyang Prescription in the Treatment of Heart Failure with Xinyang Deficiency 09 20-22
Chen Kui 2007 Effect of Wenyang Huayu Decoction on cardiac function in patients with refractory heart failure 5 8 676-677
Liu Yu 2007 Wenyang Huayu Decoction for the treatment of 48 cases of chronic congestive heart failure 20 3 19-20
Yan Xia, et al. 2005 Clinical study on the treatment of chronic congestive heart failure by warming Yang and activating blood circulation
Sun tzu.Ma Wenjing clinical Study on the treatment of chronic heart failure by warming Yang and Activating Blood Circulation 12, 5, 36-37
Effect observation on 60 cases of chronic congestive heart failure treated by warm-yang Activating Blood circulation and Water-replenishing Method 31 8 1164-1165
Shen Anming, et al. 2015 Wenyang Huoxue Lishui Recipe on the precursor of natriuretic peptide and quality of life in patients with chronic heart failure 11
Guo Meizhu;Xiao Yanqian 2011 Wenyang Huoxue Li Shui Recipe treatment of chronic congestive heart failure clinical observation 3
Xia Huiying 2005 Wenyang Huoxue Li Shui Prescription in the treatment of heart failure diuretic resistance 30 cases 10
Shi wu da, et al. 1997 Clinical and Experimental study of Wenyang Jianxin Oral Liquid in treating Congestive heart Failure 6
Li Fan 2006 Clinical observation on the treatment of chronic congestive heart failure by wenyang Lishui and Invigorating Qi and promoting Blood Circulation 15 11 1212-1213
Zheng Jun 2005 Treatment of 48 cases of chronic heart failure with Wenyangli water method 2
He Changguo 2006 Clinical observation of 50 cases of chronic heart failure treated by Wenyang And Li Shui method 18 6 560-561
Yu wen;Wu Xiuchuan;Xu Hongyan 2009 Clinical observation on the treatment of chronic heart failure with Wenyang Lishui method 5
Zhang Ming-xi 2007 Clinical study on improving the quality of life of patients with chronic heart failure by wing-Yang Li Shui Huo-xue Method 25 2412-413
Bao-ming li, et al. 2012 Wenyangqiangxin Decoction combined with Western medicine in the treatment of 30 cases of chronic heart failure
Wang Minsheng 2005 Wenyang Qiangxin Decoction for the treatment of 30 cases of chronic heart failure 10
Xu Wengang 2007 Wenyang Qiangxin Yin on the treatment of chronic heart failure in the elderly clinical study 26 9 601-602
Ping Shi; Clinical Observation of Wenyang Tongmai Prescription in the treatment of chronic congestive heart failure 22 27 2573-2574
Yan Qin. 2012 Effect of Wenyang Invigorating Qi and Activating Blood circulation and Benefiting Water on plasma BNP level in patients with chronic cardiac insufficiency 28 6,520-522
Qing-song huang;XiaoWei;Clinical observation of Wenyang Yiqi Xingshui Traditional Chinese medicine in the treatment of refractory heart failure 9
Jin-xiang li. 2006 Effect of Wenyang Invigorating Qi, Promoting blood Circulation and Benefiting Water on plasma renin system in patients with chronic heart failure 15 5 45 468
Jin-xiang li; Fang Juzheng 2006 Effect of Wenyang Yiqi Decoction on serum B-type natriuretic peptide in patients with chronic cardiac insufficiency 28 3 21

Hui-jun zhou;Yin xiaoxing;Niu Yongjun 2010 Clinical study on the treatment of Chronic congestive heart failure with Wenyang Yiqi Decoction
Zhang Jinbo;Effect of 2012 Wenyang Yiqi Yangxin Decoction on left ventricular ejection fraction and brain natriuretic Peptide in patients with chronic heart failure
Jian wang ping, et al. Effect of Wenxin Granule on cardiac function and hemorheology in chronic heart failure 5
Yutao;Ms li.Clinical observation of 37 cases of chronic heart failure treated by Wenxin Granule 26, 11, 9-10
Clinical observation of 60 cases of chronic heart failure treated by Jingchanglin 2007 Wenxin Granule 28, 4, 19
KouYaoJun;Liu Xitao 2017 Western medicine combined with Traditional Chinese medicine Yiqiwenyang Decoction for the treatment of chronic heart failure clinical efficacy observation 2 11 105-106
Ma Zhen;Xiao-jie Chen;Liu Yong 2007 Xianren Jixin Tablet in the treatment of chronic systolic heart failure clinical observation 16 3 261-262
Ying white;Jian-guo qin;Ya-hong wang;Observation on the clinical Effect of Xinkang Mixture on chronic congestive heart failure 25 9 1886-1887
Zhou Heng, et al. 2007 Clinical study on improving the quality of life of patients with chronic congestive heart failure with Xinkang Oral Liquid 29 5 394-396
Li Ruwen, et al. Clinical observation of Xinlikang Decoction in the treatment of refractory heart failure 29 2 10-11
Wu Xinyu;Liu Shu;Julliard 1997 Clinical study on the effect of Cardiomyopathy on bilateral cardiac function in patients with congestive heart failure
Wong.Yao Jing;Discussion on the clinical effect and mechanism of Xin Shu nasal drops in the treatment of chronic heart failure
Niu HuiXi, et al. Relationship between improvement of cardiac function in heart failure patients and serum levels of shenmai injection and inflammatory cytokines 19
Xu-dong xiong;The king left;Zhou Jiyan 1999 Clinical observation of Xinmaolong Injection in treating congestive heart failure 8 2 54-55
Xiao-lei Zhao. Observation on the therapeutic effect of 2017 Xinmai Long Injection on acute cerebral infarction complicated with chronic heart failure 15 1862-1864
Yuan Guili, et al. Observation on the efficacy of xinmailong Injection in the treatment of chronic heart failure 37 10 1545-1548
De-ming Deng, 2002 Xinning Decoction in treating 50 cases of congestive heart failure clinical study 8
Ze-yin Liu, et al. Clinical observation on 30 cases of chronic heart failure treated by Luo Ying no.1 formula 2007
Du Xianchun, et al. 1997 Clinical study on the treatment of congestive heart failure with Xinshuaikang 5
Zhang Xiaojiang, et al. 2006 Clinical study on The treatment of Congestive Heart failure with Xinshuiling Granule 21, 30-31
Fu Jihong 2006 Xinyuan Capsule and Astragalus Injection in the treatment of chronic systolic heart failure clinical observation 22 10 1536-1537
Wang qin, et al. 2015 The treatment of chronic congestive heart failure with sanjiao, Purgatory and phlegm, promoting blood circulation and invigorating water.
Wang qin. Study on the clinical efficacy of Daoxiefei Decoction in the treatment of congestive heart failure 29, 870-872
Zhang Junru; Ren Dezhi 2015 Yangxin Mixture treatment of chronic heart failure Clinical observation 2
QuJiaWu;Yu jianghao 2010 yangxin tablets in the treatment of chronic heart failure clinical efficacy observation 14, 15, 89,97

Qu Jiawu 2008 Clinical observation of Yangxin's syndrome in treating chronic heart failure 20 5 989-990
Liang Lanying 2013 Yangxin Tang combined with Carvedilol in the treatment of 42 cases of chronic congestive heart failure
Yao Shijun 2011 Application of Yangxin Tang in heart failure 3 19 238
Su Xianhong;Wang Suting;Mi Dan 2014 Yangxin Decoction treated 50 cases of heart failure with deficiency of Qi and Yin 3
He Xinbing, et al. 2009 Yangxin Tongmai Decoction intervenes the left ventricular diastolic function in patients with diastolic heart failure 10
Jiang Ting; Chen Zhuoyan; Efficacy observation of 2015 Yiqi Fumai injection combined with trimetazidine in the treatment of chronic heart failure 37, 499-101
Xiu-ying ren, et al. Clinical efficacy of Yiqi Huayu Granule in the treatment of chronic systolic heart failure 29 10 62-63
Gao Chen; Clinical observation on the treatment of 63 cases of chronic diastolic heart failure with Liji 2015 Yiqi Huayu Granule 13 17 1965-1967
Xiu-ying ren, et al. 2012 Yiqi Huayu Granulator in the treatment of 40 cases of chronic heart failure, 33, 2, 144-145
Sun Zhenxiang 2008 Yiqi Huayu Lishui Decoction for the treatment of chronic heart failure 29, 1, 74-75
Jiang Guangping, et al. 2014 Effect of Wang Xiaofeng's Replenishing Qi and activating Blood circulation method on prethrombotic state in 118 patients with chronic heart failure 32 2 1-4
Dan-ping li, et al. Effect of Invigorating qi and activating Blood circulation on cardiac function in patients with congestive heart failure
Gu Huan, et al. 2008 yiqi huoxue method for chronic heart failure patients TNF alpha and Ang Ⅱ effects of June 4, 381-382
Sun Boqing, 2006 Clinical study on the treatment of congestive heart failure by supplementing Qi and activating blood circulation
Xiao-hua dai, et al. 2005 Clinical observation on the treatment of congestive heart failure by supplementing qi and activating blood circulation
Huang Qing. Observation on the clinical efficacy of Wen Lihui 2011 Replenishing Qi and activating Blood circulation in the treatment of chronic congestive heart failure
Zhong Yan, et al. Effect observation of Ding Bonhan's 2017 Method of Invigorating Qi and activating Blood in treating acute attack of chronic heart failure
Peng Minqiu 2004 invigorating Qi, activating blood and removing blood stasis in treating 100 cases of chronic congestive heart failure.
Liu Huihong, et al. 2011 treatment of 148 cases of chronic congestive heart failure by supplementing Qi, activating blood circulation and benefiting water
Zhi-hua guo, et al. Clinical study on improving left ventricular function of Yi Xiang Hong 2005 Yiqi Huoxue Li Shui Prescription 1
Clinical Observation of 2017 Yiqi huoxue Li Shui Decoction combined with conventional Western medicine in the treatment of chronic heart failure 39 3 388-392
Hu Wanli 2012 Yiqi huoxue Tongmai Powder combined with Western medicine in the treatment of 40 cases of chronic heart failure
Zhou Pingsheng, et al. 2015 Clinical Observation on the treatment of chronic congestive heart failure with The method of invigorating Qi, promoting Blood Circulation, warming Yang and Benefiting Water
Zhang Li, et al. 2001 treatment of 70 cases of chronic heart failure by supplementing qi, promoting blood circulation, warming Yang and benefiting Water. 3

Liao Zhanmei 2007 Clinical observation on the treatment of refractory heart failure by Invigorating Qi and Strengthening Spleen and Heart Therapy 16 9 1051-1052
Li He, et al. 2016 Influence of Yiqi Shuxin Decoction on cardiac function and Quality of life of patients with diastolic heart failure (Heart qi deficiency syndrome.
Wu Junhua, et al. 2016 Yiqi Tongluo method on BNP and HS-CRP in patients with chronic heart failure 1
Yao Facheng 2014 Yiqi Tongmai Decoction for the treatment of 50 cases of heart failure clinical observation 27 10 200-201
Zhang Zhong, et al. 2007 Adjuvant therapeutic effect of Yi-Wen-Yang, Huoxui-Lishui on congestive Heart failure 24 3 201-204
Zhang Zhong, et al. Effects of Yiqiwenyang and Huoxue Lishui on neuroendocrine factors in patients with congestive heart failure 37 8 15-16
Chen Lihua. Clinical experience in treating chronic congestive heart failure with yiwenyang, Huoxue And Lishui therapy in 2009
Jin-hui Du, et al. 2016 Clinical Study of Yiqiwenyang, Qingrejiedu in the treatment of chronic heart failure 31 2 723-725
The influence of 2011 Yiqiyuyang method on the quality of life of patients with chronic heart failure 20, 539-540
Chen Lianzhong. 2008 Yiwenyang method in the treatment of 30 cases of chronic congestive heart failure
Feng Haibo 2006 Treatment of 102 cases of heart failure by Yiqiwenyang method
Chang Ruili. A Clinical study on the Treatment of Chronic Heart failure by Combining Yangguyuan Prescription with Western medicine 11 11 135-136
Fu Chunsheng, et al. Clinical study on the treatment of 34 cases of refractory heart failure by Yiqiwenyang Desilting method 24 12 1380-1381
Shen Jinghong. 2010 Yiqiwenyanghuayu Li Shui method combined with Western medicine in the treatment of 64 cases of refractory heart failure: 2
Shen Chengling, et al. 2003 effect of yiqiwenyang invigorating blood and Invigorating water on cytokines and oxidative stress in patients with heart failure 6
Zhu Huiping. 2008 Treatment of 100 cases of congestive heart failure by Yiqiwenyang Huoxue Li Shui method
Shi Moyi, et al. 2015 Yiqiwenyang Huoxue Li Shui Prescription for treating 40 cases of chronic heart failure 2
Gu CanLi, et al. 2009 clinical observation on the treatment of chronic heart failure by yi-wen-yang-li-shui method 18, 19,67
Observation on 60 cases of chronic heart failure treated with Yiwenyang Decoction combined with Western medicine
Clinical observation on the treatment of chronic heart failure with Yiwenyang Decoction 25, 721-723
Xu Ying;Ding Yuhui;Jin Saifei 2003 Yi Air temperature Yang Xie Fei Li Shui treatment of chronic congestive heart failure 7
Cai-li Yang, et al. 2009 Clinical observation of 36 cases of senile chronic heart failure treated with Yiqi Yangxin Tongmai Decoction combined with Western medicine
Li Shufang 2005 Effect of Traditional Chinese medicine on exercise tolerance in patients with chronic congestive heart failure 3
Shi Jianping; Zhang Jianping 2008 Clinical study of Chinese herbal Medicine for Improving Qi and Nourishing Yin in the treatment of heart failure 14 16 2544-2545
Wang Yonglin 2012 Effect of Yiqi Zhengyang Decoction on improvement of chronic heart failure symptoms and heart function 24 6 522-523
Zhang Lin 2007 Yixin Capsule treatment of 98 cases of congestive heart failure clinical study 26 4 242-243

YanLei;Xue Wenhai;Yin Lijun 2012 Clinical Study on the effect of Yixin Decoction on cardiac function in patients with chronic cardiac insufficiency 26 9 1-2

Zhai Lihuang 2004 Yiyuan Huoxue Prescription in the treatment of senile heart failure clinical study 6

Observation on the effect of Peilu Yangxin Decoction in the treatment of senile chronic congestive heart failure 13

Qi Bin 2014 To explore the effect of Zhenwuqiangxin Decoction combined with Western medicine on heart failure 21

Lou Yifei 2013 Zhenwuqiangxin Decoction combined with Western medicine for the treatment of senile Heart failure Clinical Observation 45 10 10-12

Xiao-feng zhu; Dong Debao 2005 Zhenwu Tang modified treatment of 57 cases of chronic congestive heart failure 6

Dong Debao;Zhang Ronghua 2005 Zhenwu Decoction for the treatment of chronic congestive heart failure clinical observation 4

Li Ming, et al. 2015 Zhenwu Decoction for the treatment of 49 cases of chronic heart failure clinical observation 36 207-208

Guo Fengjie, 2014 Zhenwu Tongwei Treatment of 30 cases of heart failure quality of life observation. 02 71-72

Cheng Hua, et al. A clinical study on The treatment of moderate and severe chronic heart failure with Orgasmic 2008 Zhenxin Mixture 28 11 797-798

Huang Rui 2013 Curative effect observation of 50 cases of chronic heart failure treated by Combination of Chinese and Western

Clinical study on the combination of Chinese and Western medicine in the treatment of chronic heart failure 43 6 19-20

Wu Guanlin 2013 Application of Integrated Traditional Chinese and Western medicine in rehabilitation treatment of chronic heart Failure 12 21-22

CAI Haijian 2006 Curative effect observation of combined Chinese and Western medicine in treating congestive heart failure 2

Li Jinhong 2006 Clinical observation of combined Chinese and Western medicine in the treatment of congestive heart failure 33 7 860-861

Observation on clinical Efficacy of Combined Traditional Chinese and Western medicine in treating Congestive heart failure 9 9 106-107

Yin xiaoxing. Clinical observation of 56 cases of congestive heart failure treated by integrated Traditional Chinese and Western medicine in 2011

Yan-li wang;Liu as show;Liu Zhiming 2010 Effect of combined Chinese and Western medicine on cardiac function in patients with chronic heart failure 8 10 1151-1152

Summary of the treatment of 32 cases of senile chronic congestive heart failure with integrated Chinese and Western medicine in 2011:27, 4, 14-15,57

Guo-liang chang, et al. 2013 Curative Effect observation of combined Traditional Chinese and Western medicine on senile chronic heart failure 35 5 26-27

Zhi-gang Liu. Observation on the curative effect of combined Traditional Chinese and Western medicine on 60 cases of senile Intractable heart failure in 2016 7

Sun Yuan treated 32 cases of chronic congestive heart failure with the combination of Chinese and Western medicine in 2005

Shao-feng Liu, et al. 2005 Treatment of 36 cases of chronic congestive heart failure with integrated Chinese and Western medicine observation 4

Zhong Lianjiang 2011 Combined Traditional Chinese and Western medicine in treating 38 cases of chronic congestive heart failure

Hai-cheng Zhang. Curative effect observation of 68 cases of chronic congestive heart failure treated by integrated Traditional Chinese and Western medicine 26 4 532-533

Yang Guangmei;Zhu Kun; Observation on the curative effect of combined Traditional Chinese and Western medicine on chronic congestive heart failure 20, 445-446
Fang Riming 2005 combined Traditional Chinese and Western medicine to treat 40 cases of chronic systolic heart failure 11
Feng Hongwei observation of 38 cases of chronic heart failure treated by integrated Traditional Chinese and Western medicine in 2014 30 9 851-852
Gu Canli 2009 Clinical observation of 43 cases of chronic heart failure treated by combination of Chinese and Western medicine 6
Li Shanxin;Liu Ying, 2011 Treatment of 45 cases of chronic heart failure with combination of Chinese and Western medicine: 23, 2, 47-48
Clinical observation of 82 cases of chronic heart failure treated by integrated Traditional Chinese and Western medicine 3
Wu Qi-xiang 2010 Curative Effect observation of combined Traditional Chinese and Western medicine on chronic heart failure 12 5 193-194
Han Yanqiu. Observation on the curative effect of combined Traditional Chinese and Western medicine on chronic heart failure
Chen qing. Clinical observation of 43 cases of chronic heart failure treated by integrated Traditional Chinese and Western medicine 8 10 44-45
Clinical Analysis of combined Traditional Chinese and Western medicine in the treatment of chronic heart failure 3 9 137-138
Clinical observation of 30 cases of refractory heart failure treated by integrated Chinese and Western medicine 2, 14, 81
Xu Xin 1997 Treatment of 31 cases of refractory heart failure with integrated Traditional Chinese and Western medicine 3
Wang Jixia. Clinical Observation of combined Traditional Chinese and Western medicine in the treatment of intractable heart failure 19, 7, 1220-1221
Observation of curative effect of integrated Traditional Chinese and Western medicine on refractory heart failure
Shao Nan; Gong Lihong 2014 Influence of Traditional Chinese medicine on E/A ratio and 6min walking test in patients with diastolic heart failure 16 3 100-101
Liuli, Gao He. 2009 traditional Chinese medicine, the heart Ⅰ DouJinJin clinical observation on treatment of congestive heart failure, serum concentrations of sICAM 1 and 6 min walking test the influence of 36 1 19 to 20
Zeng Jingrong Clinical observation of TCM combined with blood Purification in the treatment of Chronic Refractory heart failure 23, 12, 2379-2380
Li Xiaoxia;The party XiaoMian;Yang Sheng 2005 TCM injection syndrome differentiation for treatment of chronic congestive heart failure 1
Chen Jing;Jiang Ping;Fan Tingting 2013 Application of TCM Wenyang And Li Shui Method in the treatment of Heart failure 22 16 1751-1752
Even built Aaron;Gong Lihong 2014 Influence of Traditional Chinese medicine on 6-minute walking test and vascular pseudohemophilia factor levels in patients with diastolic heart failure 4 154-154
Lin Jianmin;Li-hong gong;Yu Bo 2013 effect of TRADITIONAL Chinese medicine on BNP and 6min walking test in patients with diastolic heart failure 8 3 258-260
Yang Ge 2013 Clinical Efficacy analysis of TCM in the treatment of chronic congestive heart failure 11 36 205-206
Tungen Chen, et al. Experience of clinical efficacy of TCM Zhenwuqiangxin Decoction combined with Western medicine in the treatment of senile Heart failure 13 12 108-109

Ke-jun Wang. A Study on the therapeutic Effect of TCM on Heart and Kidney Yang Deficiency syndrome in chronic heart failure
Zhang Huaping 2017 Juyang Tongmai Peiyuan Decoction for the treatment of 40 cases of heart failure clinical observation 15 22 196
Effect of Injection of Yiqi Fumai (lyophilized) powder on cardiac function and Plasma brain natriuretic Peptide in patients with chronic heart failure
Zhang lei.Zhang Shigui, 2013 Clinical Observation on the treatment of refractory heart failure with injection of Yiqi and fumai combined with Milinol. 22, 7, 1234-1235

Studies without definite diagnostic critetia

Author Year Title Volume IssuePages

Tian Jingcheng. Efficacy evaluation of Tian Zongwen's 2016 6min walking test on The treatment of congestive heart failure with Yiqi Huoxue Capsule 29 2 337-338

Chang Gongwei. Treatment of 48 cases of chronic heart failure by replenishing Qi, eliminating phlegm and activating blood circulation

Xu Chen, et al. 2016 Buqi Yangyin Decoction for the treatment of 92 cases of heart failure with deficiency of Qi and Yin

Wei Mengling 2012 Clinical Observation on The Treatment of Heart qi deficiency syndrome of chronic heart failure with Buxin-Qi Oral Liquid combined with Western medicine 10 11 1284-1286

Observation on the curative effect of Buyang Yiqi Decoction in treating chronic heart failure

He Xinrong;Li Nini 2016 Buyiqiangxin Tablet combined with L-carnitine in the treatment of chronic heart failure clinical study 10

Gao wei, et al. 2016 Buzhong Yiqi Wuling Decoction combined with Western medicine in the treatment of chronic heart failure clinical observation

Guo Lihong;Guo Ying;Lin Jieming;Yue Xiaojun 2005 Color Doppler ultrasound in the treatment of chronic congestive heart failure effect analysis 1

Chun-ying huang;Clinical observation of Shenfu Decoction combined with Western medicine in the treatment of chronic congestive heart failure 32 2 131-132

Wenkai zhou;Lin Shengyi;Yang Weimin 2013 Effect of Shenfu Injection on NT-probNP in patients with coronary heart disease and heart failure 22 9 1625-1626

Guo Jinjian;Yue-jin guo;Qiao Jianfeng 2006 Effect of Shenfu Injection on cardiac function and myocardial fibrosis in ischemic cardiomyopathy

GengXiaoYin, et al. A clinical study of Shenfu Injection in the treatment of Congestive heart failure 15 2 150-151

Zhang zhiguo, et al. Effect of Shenmai Ningxin Mixture on TNF- in patients with chronic heart failure 13

Li Songhe;Xie Huadong;Hu Zhu;Effect of Shenmai injection on heart failure and heart rate Variability in patients with coronary heart disease 20 35 43-44

Jun liu da;Huang Linxi effect of Shenmai injection on left ventricular diastolic function in patients with chronic heart failure 4

Yu Shicheng, et al. 2017 Shenmai Injection on left ventricular ejection and cardiopulmonary motor function in ischemic cardiomyopathy

NiuHuiXi;Huaning;Qizhi 2005 Shenmai injection improved cardiac and immune function in patients with congestive heart failure

Hui-ligan;Tian Xiao-yuan 2006 Efficacy of Shenmai injection in the treatment of left ventricular dysfunction after coronary artery bypass grafting 30 3 227-228

Chen Shucun;Peng Shutao;Li hui.Influence of Li Bo 2014 Shenyu Yangxin Decoction on 6-minute walking test in patients with chronic congestive heart failure 41 10 2111-2113

Li hui.Yan-ping sun;Clinical evaluation of Chen Tong in the treatment of congestive heart failure by Shenmai Injection and Fasudil 4

Hu Wanying;Chen Zhaochuan 2006 Theoretical and clinical study of Treating Congestive Heart failure with Qiangxin Mixture based on Kidney Theory

Chen Yunlu;Wen-jun huang; Effect of Salvia miltiorrhiza 2004 on oxidative/antioxidant system in elderly patients with refractory heart failure 4

Xu Shule; Therapeutic Effect observation of 2014 Fuzheng Yangxin Decoction in the remission stage of chronic heart failure 12 18 55-56

Rong Tangli. Effect of Compound Danshen dropping pills on plasma CGRP and ET concentrations in patients with coronary heart disease complicated with congestive heart failure 7

Mei liang, et al. 2006 Clinical study on the effect of puerarin on BNP level in patients with chronic heart failure 26, 12, 17-18

Yao Baojie; Effect of 2014 Guicao Xinyangfang on cardiac function of elderly patients with chronic heart failure 3

ChanJinPing; Yang Guofeng 2014 Huayu Lishui Decoction for the treatment of 49 cases of chronic congestive heart failure clinical observation 12 8 174

Xu Huicong, et al. 2011 Astragalus Injection on TCM symptoms and Syndrome types in patients with acute decompensated chronic heart failure 43 2 22-24

Minghua Jiang, et al. 2008 Multi-parameter analysis of Huoxue Fumai Decoction for heart failure in ischemic cardiomyopathy 35 7 961-963

Gao Deju;Wang Yumin;Su Yulun 2014 Huoxue Qiangxin Decoction for the treatment of chronic heart failure clinical efficacy 32 7 104-105

Zhang Shuangwei 2012 Jiawei Shenfu Granule in the treatment of chronic heart failure clinical study 44 12 20-21

Huang Yunsheng;Luo Renhan;Clinical study of Xu Kai 2009 Modified Sini Decoction in the treatment of chronic heart failure

Xiaofen Ruan, et al. Effects of Kanli Decoction on activity tolerance, quality of life and aggravation frequency of heart failure in patients with chronic heart failure

Wei Jianhua. 2015 Carvedilol combined with Shiwei Qiangxin Decoction in the treatment of chronic heart failure with Deficiency of Heart and kidney Yang 5

Yong-ji li;Clinical analysis of Ling GUI Zhugan Decoction in the treatment of congestive heart failure 10 23 178-178

Zhang Jianfeng 2012 Ling GUI Zhugan Decoction for the treatment of chronic heart failure effect observation 10, 16, 84

Cai Hui, et al. 2006 Quantitative assessment of improvement of cardiac function in patients with congestive heart failure by Lu Jiao Fang 10 27 152-155

Cai Hui, et al. 2006 Effect of Lujiao Prescription on improving left ventricular hypertrophy in patients with congestive heart failure 10 43 222-224

Xu Guangzong 2011 Clinical efficacy observation of oral medication once a day in the treatment of chronic heart failure 17 7 17-18 113

GengHuaPei. 2017 Clinical analysis of Bilicong Metoprol combined with Shensong Yangxin Capsule in the treatment of chronic cardiac insufficiency 39 14 2149-2151

Jian-bo Yang;Managed;Wang Xiaofeng 2009 Clinical observation of Ningxin Tongbi prescription in treating chronic heart failure 3

Analysis of the curative effect of Ningxin Tongbi Capsule combined with rehabilitation treatment on heart failure with mutual obstruction of phlegm and blood stasis 9 14

AnYanXia;Bowie cheung.Ding Zhixiao 2010 Qi Shen Yi Qi Dropping Pills in the treatment of coronary heart disease heart failure clinical observation 18 3 367-368

Wang Qingquan clinical Observation of 2016 Qishen Yiqi dropping Pill in the treatment of CHD heart failure 35 17 36

Observation of clinical efficacy of Qishen Yiqi dropping Pills in the treatment of CHD heart failure 10 16 103-104

Yang Guodong 2015 Qishen Yiqi dropping Pills in the treatment of chronic congestive heart failure clinical analysis 11 7 114-115

Xin Fanyong 2015 Qishen Yiqi dropping Pills in the treatment of 40 cases of chronic heart failure
Li Yanzi;Yan Xuehuai 2015 Qishen Yiqi dropping Pills in the treatment of chronic heart failure clinical efficacy 4
Observation on clinical effect of 2017 Qishen Yiqi dropping Pill in treating chronic heart failure 15 2 173-174
Wang Jianxiang 2012 Qishen Yiqi dropping Pills in the treatment of chronic heart failure clinical efficacy observation 10 8 901-902
Lan Jun 2012 Clinical Observation of Qishen Yiqi dropping Pill in the treatment of heart failure patients with ischemic cardiomyopathy 18 14 77-78
Wang Xiaofeng 2017 Influence of Qi Hong Powder on patients with chronic heart failure syndrome of qi deficiency and blood stasis 07 3292-3295
Cui Lingling;Zhang Heyan 2012 Qiliqiangxin Capsule in the treatment of 34 cases of chronic heart failure clinical observation 34 6815-818
Yang Hongtao 2012 Qiliqiangxin Capsule in the treatment of 50 cases of chronic heart failure clinical observation 28 5 21-22
Cui Xinzheng 2014 Qiliqiangxin capsule treatment of chronic heart failure clinical efficacy observation 10 273-273,274
YanHuiFang;Cao Zhe;Clinical efficacy of qiliqiangxin Capsule in the treatment of 50 cases of Heart failure with Xinshenyangdeficiency 35 8 74-75
Sun Lanjun, et al. 2006 Clinical observation and analysis of Qiangxin Granulation in the treatment of chronic congestive heart failure 25 3 146-148
Chen Zhaoshan;Yao-rong dong;Clinical effect of Qiangxin Mixture on chronic congestive heart failure 1
Huang Lin 2003 The effect of Qiangxin Granule on ventricular systolic and diastolic function in patients with chronic heart failure 4
Yang Sujuan 2011 Qiangxin Granule in the treatment of 49 cases of chronic heart failure: 26 3 504-505
Lin Xiatian. 2016 Randomized controlled study of Qiangxinli Water Prescription on B-type natriuretic peptide and left ventricular end-systolic volume in patients with chronic heart failure with Spleen-kidney Yang deficiency
Wang Yakuan;Ding Zhixin;Bai Xiaoyan, 2014 Clinical Study of Qiangxinningshuai Oral Liquid in the treatment of chronic congestive Heart Failure 23, 4 682-683
WenZhiHao, et al. 2014 Study on the effect of Qiangxin Tang on exercise Tolerance in patients with chronic heart failure
Wang Changyu;Observation on the efficacy of Qiangxin Decoction in the treatment of heart failure patients ii
Yu Xiaoping 2007 Qiangxin Decoction for the treatment of heart failure effect Observation 16 3 263-264
Xiaodong wang;Xiao-yan Yang;A clinical study of Feng Hui 2011 Qiangxin Liquid in the treatment of chronic congestive heart failure
Xiao-dan zheng.Observation on the curative effect of Qiangxinyin on 75 cases of heart failure caused by Deficiency of Heart and kidney Yang
Xiao-dan zheng.Ding Yingchun 2014 Qiangxinyin for the treatment of 75 cases of heart failure with Xinshenyang-deficiency 01 24-25
Qu Zhengyan, et al. 2012 Qinlian Xiangsha Liujunzi Decoction for the treatment of 30 cases of phlegm-turbidity chronic obstructive heart failure clinical observation 28 6 1057-1058.
Cai Guang, et al. 2003 Therapeutic effect of panax notoglucoside on heart failure in ischemic cardiomyopathy 2

Huang Jihua 2004 Musk Heart-protecting Pills in the treatment of coronary heart disease with cardiac insufficiency
Jing-yuan MAO, et al. Study on the mechanism of Shengmai injection in the adjuvant treatment of congestive heart failure 5
Yang Xiaoqun, et al. 2002 Shengmai Injection in the treatment of 50 cases of senile congestive heart failure 11
Wang Jingong, et al. 2008 Ten Flavour Qiangxin Decoction for the treatment of Congestive Heart failure clinical Study 24, 9, 11-13
Wang Yanmin clinical observation of sini Decoction in the treatment of 72 cases of Heart failure caused by Yang Deficiency 31, 4520-521
Wang Ma, et al. 2017 Clinical study of Harmonic Yingwei Method on cardiac autonomic nervous dysfunction in chronic heart failure 28 6 1380-1382
Zi Yong, et al. Effect of Tongguan Capsule 2011 on cardiac function and serum SDF-1 in patients with coronary heart disease after interventional therapy 43 8 5-7
Wang Wenqing 2009 Tongluo Prescription for the treatment of 125 cases of heart failure with coronary atherosclerotic heart disease 31 8 1148-1149
Xu Pinghe 2012 Tongmai Dixian Pill auxiliary treatment of ischemic cardiomyopathy heart failure observation 19 1 63-64
Peng Xiaoping. 2016 Clinical Study of Tongmai Yangxin Tang on prevention of recurrence of heart failure with Heart and Kidney Yang Deficiency 22 16 70-72
Ma Liping;Feng-lan wang;Wang Mei-ling 2003 Effect of Tongxinluo on cardiac function and exercise tolerance in patients with coronary heart failure
Zhang Xiaocui, et al. Clinical study of Li Jingrui 2005 Tongxinluo Capsule on thyroid hormone levels in patients with coronary heart disease and heart failure 3
Wei Xinghong, et al. 2015 Tongyang Huoxue Decoction for the Treatment of chronic heart failure with Deficiency of The heart and kidney 7 6 102-103
Jianbo Yang, et al. 2011 Clinical study on the treatment of heart failure with Phlegm-stasis Syndrome 34 7 669-673
Wang Zhibin;Hao Xin 2013 Influence of Wenshenhuayin prescription on 6min walking test in patients with yang-deficient water type heart failure 11 24 19-20
Gong Jiwen observation of clinical efficacy of 2016 Wenshenli Water Rescue Heart Soup in the treatment of refractory heart failure patients 6
Zhang yan, et al. 2008 Effect of Wenxin Decoction on serum brain natriuretic peptide level in patients with chronic heart failure and its curative effect Analysis 31 5 564-566
Cai Yanma, et al. 2015 Clinical study of Wenyang Prescription in the treatment of chronic heart failure 31, 15, 5-7
Liu Guoan. Observation on the efficacy of Wenyang Huoxue Prescription in treating heart failure 10 17 24-25
Zhan Ping, et al. 2016 Warm Yang invigorating blood and Water therapy on serum N-terminal brain natriuretic peptide and left ventricular function in heart failure patients with normal LEFT ventricular ejection fraction 4
Wu Tong, et al. 1997 Clinical summary of Wen Yang Jian's mental Therapy for Heart failure with Yang Deficiency 3
Kuang Kaian, et al. 2005 Clinical observation of 35 cases of cardiac insufficiency treated with warm Yang, beneficial qi and activating Blood circulation method 2
Wu Hao 2016 Wenyang Lishui prescription combined with Western medicine in the treatment of refractory heart failure in 30 cases 22
Chen;Zhou Xinhong 2011 Wenyang Li Shui Huoxue Prescription for the treatment of 110 cases of chronic congestive heart failure 20 2 313-314
Sun Shengmei, et al. 2015 Clinical observation of Wenyang Lishui Capsule combined with Western medicine in the treatment of chronic heart failure 03 400-401+403

Zhang Maogen 2010 Wenyang Tongluo Huoxue Prescription in the treatment of chronic heart failure clinical observation 4
Ren Jie 2014 Wenyang Yiqi Huoxue Prescription in the adjuvant treatment of ischemic cardiomyopathy heart failure observation 27, 246
Jing En 2014 Effect of Wenyang Yiqi Yangxin Decoction on plasma brain natriuretic peptide and Cardiac Function in patients with chronic heart failure
Xiao Junjie, 2014 Wenyang Yixin Decoction for the treatment of 38 cases of chronic heart failure: 22, 17, 115-116
Li Fengling, 2017 Western medicine combined with Wenyang Buxin Decoction for the treatment of chronic heart failure (systolic) and analysis of the effect on brain natriuretic peptide level 36 14 79-80
Xu Hongshan, et al. Clinical effect of Xiefei Lishui Prescription in the treatment of heart failure and its effect on myocardial cell endoplasmic reticulum stress-related apoptosis indons 35 5 82-83
Liu Zhongbin, et al. 2007 Xingong Rehabilitation Drink for the treatment of 96 cases of cardiac insufficiency 9 17 109
Fan Quan, et al. Influence of Lin Jing on serum adiponectin level and cardiac function in patients with chronic heart failure by Xinmailong Injection 2017
Di Jie, et al 2014 Xinmai Long Injection combined with Qishen Yiqi Dropping Pill in the treatment of coronary heart disease heart failure clinical study ii
Jin Yu 2014 Heart Failure Mixture treatment of 30 cases of chronic heart failure 19
Geng Ping;Yang Yuling;Wu Bin 2014 Heart failure Mixture treatment of 40 cases of chronic heart failure 33 30 13-14
Tian Li; Hui Junsun Effect of Yu Hong 2000 Xinshukang Granule on cardiac function parameters of echocardiography in patients with cardiac dysfunction 10
Li Hua. 2007 Effect of Huang Bin Xinshuikang Granules on serum MMP-2 and TIMP-2 levels in patients with heart failure after myocardial infarction 20 2 37-38
Song Qingqiao, et al. 2002 Heart failure in patients with chronic congestive heart failure angiotensin Ⅱ, the effect of aldosterone.
Chen Xumin, et al. Observation on the correlation between plasma BNP level and combined TCM and WESTERN medicine in the treatment of Heart Failure 1
Yang Wei. Clinical observation and Basic Research of yangxinjianshuai Decoction in treating chronic mild to moderate congestive Heart failure with Kidney-Yang Deficiency.4
Sun Fujun, et al. Clinical observation of Nourishing heart and Detoxifying method in treating chronic congestive heart failure 8
Efficacy of Yangxin Yiqi Decoction combined with Western medicine in the treatment of chronic heart failure 20, 9119-120
Li Chenghui. The clinical effect of Yiqi Fumai injection on chronic heart failure 8
Li Jingfu 2015 Yiqi Fumai Injection combined with Western medicine in the treatment of chronic heart failure clinical efficacy observation 9 16 167-169.
Wang Min 2010 Yiqi Fumai Injection in the treatment of ischemic cardiomyopathy patients with heart failure clinical efficacy 16 27 47-48
Clinical efficacy and safety evaluation of Yiqi Huayu Granule in the experimental treatment of chronic systolic heart failure 21 2 334-336
Treatment of 102 cases of chronic systolic heart failure with qi deficiency and blood stasis syndrome by Yiqi Huyu Granule
Yang Baoyuan, et al. 2011 Treatment of 120 patients with chronic systolic heart failure with qi deficiency and blood stasis syndrome by Yiqi Huayu Chongji Clinical Observation

Du Xiaojie, et al. 2016 Yiqi Huayu Granule for the treatment of 60 cases of chronic diastolic heart failure with qi deficiency and blood stasis
Shi Qiushi, et al. 2012 Invigorating Qi and activating Blood circulation, wenyang and Li Shui Method combined with Western medicine in the treatment of 50 cases of chronic congestive heart failure
Kai-xuan Lin;Chen Yi 2010 Clinical observation on improvement of left ventricular ejection fraction and brain natriuretic peptide in patients with heart failure by supplementing qi and activating blood Circulation
Yuan Tianhui, et al. 2012 Effect of Supplementing Qi, activating Blood and Resolving Phlegm prescription on 6min walking test and Quality of life in elderly patients with diastolic heart failure.
He Benhong 2012 Clinical Observation of Huoxue Capsule in treatment of chronic heart failure 34 6 3-4
Huang Jingyuan, et al. Observation on the curative effect of Wan Xiaoqing 2016 supplementing Qi, activating Blood and Benefiting Water in the auxiliary treatment of heart failure. 04 282-284
Liu Qing 2015 Influence of Yiqi huoxue Prescription on improvement of cardiac function indexes in patients with coronary heart disease and Heart failure.
Meng Xianliang 2016 Yiqi Huoxue Li Shui Mixture on the clinical indicators of heart failure patients 31 13 1901-1902
Dong Bo, et al. 2012 Effect of Yiqi Qiangxin Decoction on plasma BNP and 6min walking distance in patients with chronic heart failure 30 12 2740-2742
Zhang Li 2011 Yiqi Qiqiang Xin Yin in the treatment of 40 cases of CHD heart failure 25 7 58 85
Wen Tiansheng 2015 Yiqi Tongyang Fumai treatment of 43 cases of chronic congestive heart failure 13
Huang Xuan;Jing-yuan MAO;Observation on the therapeutic Effect of Yiqiyang and Yiqi nourishing Yin in treating chronic heart failure.
Wu Zhian;Wang xiaofeng;Chen Jihong 2006 Clinical study on the rehabilitation effect of Yiqiwenyang method on chronic heart failure 38 9 39-40
Liang Yong, 2014 Clinical effect observation of Yiqiwenyang method in treating chronic heart failure 21, 3, 98-99,102
Cheng Xiao yu, et al. 2007 Effect of Yiqiwenyang huoxue on neuroendocrine in patients with chronic congestive heart failure
Wang Shaobing. 2011 Clinical Observation of Yiqiwenyang Huoxue Li Shui Prescription in the treatment of chronic heart failure 3, 22, 53-54
Effect of Yiqiwenyang Decoction on cardiac function of patients with chronic heart failure. 2014 23 2 176-177.
Clinical Observation of yiwenyang Decoction in the treatment of 50 cases of Heart failure with Deficiency of Heart and Kidney 2
Cao Yun 2017 Yiwenyang Traditional Chinese medicine in the treatment of diastolic heart failure clinical study 36 10 79-80
Guo Xuejun clinical Observation of 2017 Yiqi Xiefei Granule in the treatment of chronic heart failure 12, 6 1302-1305
Xue Ruping; Zhang Lei 2014 Yiqi Yangxin Prescription combined with Western medicine in the treatment of 80 patients with chronic heart failure.
Effect of Yiqi Yangxin Decoction combined with Western medicine on cardiac function, LVEF and E/A in patients with chronic heart failure.

Lilin, et al. 2015 Improving the quality of life of patients with acute exacerbation of chronic heart failure by Yiqi Yangyin Method. 12 22 113-115 127
Li Qinghai 2013 Treatment of 30 cases of chronic heart failure 4
Tang Haijun 2016 Study on the therapeutic effect of Invigorating Qi, Nourishing Yin and Activating Blood circulation on chronic heart failure
Effect observation of Meng Xianliang's 2016 kidney-invigorating and blood-activating method in the treatment of heart failure with normal left ventricular ejection fraction
Shao Peng, et al. Clinical efficacy of Yixin Tongluo Decoction in the treatment of coronary heart disease and its influence on n-terminal brain natriuretic peptide Precursor 12
Analysis and Evaluation of efficacy of Wenyang Li Shui method in the treatment of chronic heart failure 10 22 181-183
Wang Chongjian's clinical experience of applying TCM syndrome differentiation scheme in the treatment of Heart failure 8
Wang Cailing, 2014 Therapeutic Effect observation on ischemic cardiomyopathy complicated with Heart failure by Fumai Injection
Zhou Jianqiang 2017 Zhenwuqiangxin Decoction combined with Western medicine in the treatment of senile heart failure clinical efficacy experience 36 5 49-50
Wen Yongsheng 2013 Clinical Study on the Treatment of Acute cardiac insufficiency with Integrated Traditional Chinese and Western medicine 55, 44,51
Li Guangxun, 2015 Clinical efficacy of integrated Chinese and Western medicine in the treatment of 80 cases of CHD heart failure 7, 15188-189
Clinical observation of 37 cases of congestive heart failure treated with integrated Traditional Chinese and Western medicine 17 5 107-108
Li Guowu 2012 Clinical observation of 30 cases of acute myocardial infarction complicated with left heart failure treated with combination of Traditional Chinese and Western medicine
Discussion on the clinical effect of combined Traditional Chinese and Western medicine in treating severe heart failure in emergency department 36 12 64
Observation on the clinical effect of combined Traditional Chinese and Western medicine in treating senile chronic heart failure 22 12 1733-1735
Zhang Shuyong;Effect of combined Chinese and Western medicine on serum Uric acid in the treatment of chronic congestive heart failure
Mu Xiaojing, 2006 Clinical Observation of combined Chinese and Western medicine in the treatment of chronic congestive heart failure ii
Li Huanying; Fan Limin 2014 Combined Chinese and Western medicine treatment of chronic cardiac insufficiency study 13, 8, 332
Jin Congxiang 2013 Clinical observation of combined Chinese and Western medicine in the treatment of chronic heart failure 25, 252-253
Observation on the Curative effect of Combined Traditional Chinese and Western medicine on chronic heart failure 31 8 1157-1158
Wei Nahan. Clinical observation of 50 cases of chronic heart failure treated by integrated Traditional Chinese and Western medicine 31, 9, 29
Liu Pengyun, et al. Clinical Observation on the treatment of diastolic heart failure with the combination of Chinese and Western medicine 27, 1, 24-26
Peng Xiaoping;Summary of combined Chinese and Western medicine in treating 48 cases of heart failure with Heart and Kidney Yang deficiency 2
Gao Huanping 2017 Qiangxin Decoction for the treatment of 60 cases of chronic heart failure Clinical observation 36 13 15-16
Ji-hong Chen;Wang Xiaofeng 2005 Curative effect observation on rehabilitation of congestive heart failure with Traditional Chinese medicine 3

Ji-hong Chen;Wang Xiaofeng 2005 Curative effect observation on rehabilitation of Congestive heart failure with Traditional Chinese medicine 03 13-14
Liu Guoguo 2015 Clinical Observation of TCM Zhenwuqiangxin Decoction combined with Western medicine in the treatment of senile Heart failure 7 30 187-188
Yang Wanquan, et al. 2017 Clinical efficacy of TCM Zhenwuqiangxin Decoction combined with Western medicine in the treatment of senile heart failure experience 24, 2, 49
Analysis of the clinical efficacy of TCM in treating chronic heart failure of coronary heart Disease 11
Feng Chen 2013 Clinical Observation on the treatment of chronic heart failure with coronary heart disease by injection of Yiqi Fuming Pulse (lyophilized) 28 8 1607-1608
Zhao Xinfeng;Liu Jiayun;Clinical observation on the therapeutic effect of injection of Buyiqi for treating chronic heart failure 44 13 1533-1535.

Studies with irrelevant confounding factors

Author Year Title Volume IssuePages

Yang Lan, et al. 2013 Study on the effect of Hutanquyu Prescription on BNP in patients with coronary heart disease diastolic heart failure 40 6 1163-1164

Xuemin Huang; Meng Sizhou 2005 Tongmai Yuxin Decoction for improving left ventricular diastolic function of coronary heart disease clinical observation 4

Wang Xiaojing, 2013 Clinical Efficacy observation of Wenxin Granule in the treatment of CHD heart failure 33

Wang wei, et al. 2013 Influence of Supplementing Qi, removing stasis and Resolving phlegm on diastolic function of left ventricle of coronary heart disease.

Effect of Yiqi tongluo method on left ventricular function of coronary heart disease: a clinical trial of 120 cases 5

Clinical observation of combined Traditional Chinese and Western medicine in the treatment of severe heart failure with coronary atherosclerosis 21 7 1192-1194

Zhou Haixing. 2010 Clinical Observation of Combined Traditional Chinese and Western medicine in the treatment of chronic cardiac insufficiency 30 10 873-874

Ze’en Li 2006 Clinical study of different doses of Shenfu injection in the treatment of acute myocardial infarction (ST elevation) complicated with left heart failure

Pu Quanzhou; Lu-ming Yang. 2006 Effect of Puerarin injection combined with Astragalus injection on 110 cases of ischemic heart disease with heart failure. 13(1), 38-40.

Sun Xinqiang 2017 Effect of Yiqi Tongmai Decoction combined with phenolamine on ventricular remodeling in patients with coronary heart disease and heart failure. 26, 14.

Hu Youzhi, et al. 2005 Clinical study of Qiangxin Capsule in the treatment of chronic congestive heart failure 9

Qing Li 2013Clinical observation on the treatment of 30 cases of chronic heart failure with Shengxin Decoction. 45(3):25-30.

Yucheng Su, et al. 2006 Treatment of diastolic dysfunction in patients with coronary heart disease by Songling Xuemakang. 33(14), 74.

Hu Zaiping. 2010 Effect of Wenxin Granule on heart failure of coronary heart disease.27(3):46-47.

Liu Wenju, et al. 2007 Qiliqiangxin capsule on ischemic cardiomyopathy patients movement tolerance and quality of life influence. 13(8):885-887.

Mengfen Hu, et al. 2014 Evaluation of therapeutic effect on ischemic diastolic heart failure by supplementing qi and activating pulse (lyophilized). 4(16), 7-11

Shan Jiang. 2002 The effect of Astragalus injection on coronary heart disease with systolic dysfunction was observed by impedance cardiography. 6

Yiling Pu, et al. 2013 Yuan's Yangxin Oral Liquid for treating 78 cases of chronic systolic heart failure with Coronary heart disease. 26(3), 8-10

Ingle L;Shelton R J;Rigby A S; et al. 2006 6min walking test for the repeatability and sensitivity of elderly patients with chronic heart failure

AnHaiYing; Hou Yajun; Clinical observation on The treatment of congestive heart failure by Yiqiwenyanglishui Decoction from Chamomilla 2007 26 8 493-495

Hongji An 2015 Discussion on the emergency clinical treatment of 40 patients with acute left heart failure

Cai Aining; et al. 2012 Effect of gas metabolism exercise test on rehabilitation therapy for heart failure patients 21 5 550-552

Cai Jing, et al. 2003 Astragalus injection in treating 56 cases of pulmonary heart disease and heart failure.

Guoliang Chang, et al. 2011 Observation on the efficacy of Wenyang Fuwu Decoction in the treatment of senile chronic heart failure.
Bishan Chen 2016 Analysis on the clinical effect of TCM acupoint application in the prevention of constipation in patients with chronic heart failure 13.

Duplicated report

Gong Lihong, Zhang Yan. 2012 Clinical study on the intervention of Qiangxintongmai Granule in chronic heart failure. 21(5), 691, 748
